# Distribution and Genetic Lineages of the *Craspedacusta sowerbii* Species Complex (Cnidaria, Olindiidae) in Italy

**DOI:** 10.3390/biology13040202

**Published:** 2024-03-22

**Authors:** Massimo Morpurgo, Federico Marrone, Francesca Ciutti, Cristina Cappelletti, Samuel Vorhauser, Renate Alber, Matteo Dossena, Nico Salmaso, Diego Fontaneto, Luciano Caputo, Luca Vecchioni

**Affiliations:** 1 Museum of Nature South Tyrol, Via Bottai 1, 39100 Bolzano, Italy; 2 Department STEBICEF, University of Palermo, Via Archirafi 18, 90123 Palermo, Italy; luca.vecchioni@unipa.it; 3 NBFC (National Biodiversity Future Center), Piazza Marina 61, 90133 Palermo, Italy; nico.salmaso@fmach.it (N.S.); diego.fontaneto@cnr.it (D.F.); 4 Technology Transfer Centre, Fondazione Edmund Mach, Via E. Mach 1, 38098 San Michele all’Adige, Italy; francesca.ciutti@fmach.it (F.C.); cristina.cappelletti@fmach.it (C.C.); 5 Biological Laboratory, Agency for Environment and Climate Protection of the Autonomous Province Bolzano South Tyrol, Via Sottomonte 2, 39055 Laives, Italy; samuel.vorhauser@provinz.bz.it (S.V.); renate.alber@provinz.bz.it (R.A.); matteo.dossena@provincia.bz.it (M.D.); 6 Research and Innovation Centre, Fondazione Edmund Mach, Via E. Mach 1, 38098 San Michele all’Adige, Italy; 7 National Research Council, Water Research Institute (CNR-IRSA), Largo Tonolli 50, 28922 Verbania Pallanza, Italy; 8 Instituto de Ciencias Marinas y Limnologicas, Facultad de Ciencias, Campus Isla Teja, Universidad Austral de Chile, Valdivia 5090000, Chile; luciano.caputo@uach.cl

**Keywords:** Hydrozoa, freshwater jellyfish, invasive species, genetic lineages, COI, environmental DNA

## Abstract

**Simple Summary:**

The genus *Craspedacusta* comprises invasive freshwater jellyfish species present in all continents except Antarctica. Due to the morphological plasticity of the medusa stage, the number of species in the genus *Craspedacusta* is still disputed. Here, we shed new light on the distribution of the genetic lineages of these non-native species across the Italian peninsula, Sicily, and Sardinia. Since the first Italian record in 1946 and up to the last available review in 2017, *Craspedacusta* medusae were reported in 40 Italian water bodies. In the present study, we report 21 new records of *Craspedacusta* medusae presence since its latest finding in 2017. Furthermore, we present results of the molecular analyses conducted on the collected medusae. Our findings show the presence of two distinctive genetic lineages of *Craspedacusta* in Italy: (i) a group whose distribution ranges from central to northern Italy; and (ii) a group that comprises three populations from northern Italy and the single Sicilian population known to date.

**Abstract:**

Olindiid freshwater jellyfishes of the genus *Craspedacusta* Lankester, 1880 are native to eastern Asia; however, some species within the genus have been introduced worldwide and are nowadays present in all continents except Antarctica. To date, there is no consensus regarding the taxonomy within the genus *Craspedacusta* due to the morphological plasticity of the medusa stages. The species *Craspedacusta sowerbii* Lankester, 1880 was first recorded in Italy in 1946, and until 2017, sightings of the jellyfish *Craspedacusta* were reported for 40 water bodies. Here, we shed new light on the presence of the freshwater jellyfishes belonging to the genus *Craspedacusta* across the Italian peninsula, Sardinia, and Sicily. First, we report 21 new observations of this non-native taxon, of which eighteen refer to medusae sightings, two to environmental DNA sequencing, and one to the finding of polyps. Then, we investigate the molecular diversity of collected *Craspedacusta* specimens, using a Bayesian analysis of sequences of the mitochondrial gene encoding for Cytochrome c Oxidase Subunit I (mtDNA COI). Our molecular analysis shows the presence of two distinctive genetic lineages: (i) a group that comprises sequences obtained from populations ranging from central to northern Italy; (ii) a group that comprises three populations from northern Italy—i.e., those from the Lake Levico, the Lake Santo of Monte Terlago, and the Lake Endine—and the single known Sicilian population. We also report for the first time a mtDNA COI sequence obtained from a *Craspedacusta* medusa collected in Spain.

## 1. Introduction

The freshwater jellyfish *Craspedacusta sowerbii* Lankester, 1880 (Hydrozoa: Olindiidae) was described from a water lily tank in Regent’s Park, London, England in 1880 [1]. As with other hydromedusae, *Craspedacusta* has a metagenetic life cycle with two reproductive phases: asexually reproducing polyps and sexually reproducing medusae [2]. The polyps do not have tentacles and their size ranges approximately from 0.5 to 2 mm. The medusae, instead, have an umbrella up to 20 mm or more in diameter. The species belonging to the genus *Craspedacusta*, presumably native to China [3], are nowadays widely spread in all continents except Antarctica [4,5,6]. As for many other aquatic invasive species, the dispersal of *Craspedacusta* mainly occurs through imported decorative water plants, traded pet animals, and restocked fish populations [7].

The detection of hydromedusae is troublesome. Blooms, although common, are irregular and unpredictable, and occur mostly from July to October when optimal water temperature for pelagic jellyfish development is reached [8]. Moreover, their short lifespan often hinders the sightings of these hydrozoans [8]. The presence of the polyp stage is often overlooked because these go easily unnoticed in standard sampling procedures [9]. Only a few studies report successful sampling of polyps [10,11,12,13,14]. For these reasons, Blackman et al. [15] suggest that environmental DNA (eDNA) analyses can facilitate the detection of *Craspedacusta*. In fact, in 48 samples out of 92 sites routinely sampled throughout Switzerland with kick nets, the freshwater jellyfish was detected only by mean of environmental DNA analysis. *Craspedacusta* polyps were not found in kick net samples, possibly due to their small size and inconspicuous morphology. In Korea, using environmental DNA analyses among 12 survey points in the Miho River system, mtDNA COI of freshwater jellyfish was detected in eight points [16].

*Craspedacusta* individuals are not just hard to detect in their environment; due to the morphological plasticity of their medusa stages, there is also no consensus on the taxonomy within the genus. To date, the number of valid species within the genus is still an object of dispute [6]. Molecular studies suggest the existence of at least three distinctive *Craspedacusta* lineages of putative species rank: “*sowerbii*” Lankester, 1880; “*kiatingi*” Gaw & Kung, 1939; and “*sinensis*” Gaw & Kung, 1939 [13,17,18,19,20,21]. At least two lineages of *Craspedacusta sowerbii* invaded Central Europe [12,14,22] and Italy [13,20], whereas worldwide, mtDNA COI analyses showed the presence of at least three main *Craspedacusta* lineages [21,23,24,25]. In Italy, *Craspedacusta* was first recorded in 1946 in a tank at the University of Rome [26], and further sightings have been reported for various natural and artificial aquatic habitats [27]. The latest knowledge about its distribution in Italy dates to 2017, when Ciutti et al. [28] reported their presence in 40 water bodies.

The aims of the present study are twofold: 1. to update our knowledge on their distribution in Italian inland waters; and 2. to investigate which *Craspedacusta* genetic lineages occur in Italy.

## 2. Materials and Methods

### 2.1. Review of the Available Literature Data

Targeted bibliographic searches were conducted to evaluate the presence of *Craspedacusta* jellyfishes in Italy by means of Google Scholar, SCOPUS, and ResearchGate. We used data derived from international scientific journals, the grey literature, technical reports, and newspaper articles. Furthermore, following Marchessaux et al. [7], to track citations and sightings of *Craspedacusta* on various platforms (i.e., Google, iNaturalist, and social networks such as Facebook and YouTube) we searched for the keywords “freshwater jellyfish”, “*Craspedacusta sowerbii*”, “*Craspedacusta*”, “freshwater jellyfish Italy”, and “*Craspedacusta sowerbii* Italy”. Searches were conducted in Italian and English languages. Finally, we collected direct observations from national scientific societies (such as Centro Italiano Studi Biologia Ambientale, www.cisba.eu accessed on 31 January 2024). Museums of natural sciences were asked for sightings of freshwater jellyfish and specimens stored in museum zoological collections. In addition, many records are the result of citizen science; in fact, several people, having read our previous publications on freshwater jellyfish in Italy, wrote to the authors to report their sightings, attaching photos and videos.

We gathered available geographical coordinates (WGS 84) for each site; when the coordinates were not available, these were inferred from the description of the location. Sighting locations were showed on a map grouped by 25 km × 25 km UTM grids as the centroid of the cell.

The data of occurrence records are also available as a GBIF dataset [29].

### 2.2. Field Samplings and Molecular Identification of the Novel Samples

Novel samplings were carried out opportunistically in the frame of the routine sampling activities carried out by the authors of the present work. In addition, an attempt to collect fresh samples was performed in those water bodies where the occurrence of freshwater jellyfishes was signaled by colleagues or amateur zoologists. *Craspedacusta* samples were collected by means of glass jars or zooplankton hand nets, and then fixed in situ in high-percentage ethanol. In the larger water bodies, samples were collected in the open waters from small vessels or by scuba diving. In addition to the samples collected in Italy, a *Craspedacusta* sample from Canelles Reservoir (northern Spain) was investigated as comparative material.

The gender of the medusae collected in Lake Levico (n = 2) in October 2022 and Lake Santo of Monte Terlago (n = 17) in September 2023 was determined by examining fresh gonadal tissues under a microscope at magnification 100× and 400× as reported in the literature [30,31].

*Craspedacusta* DNA was obtained from direct DNA extraction from tissues, and from eDNA samples. Sequences of the mitochondrial gene encoding for the cytochrome c oxidase subunit I (mtDNA COI) were then amplified as described below. Specimens of the medusa life stage (n = 11) were collected from five lakes and two reservoirs, whereas eDNA sampling was performed in three other lakes (see Table 1 for further details).

Specimens of *Craspedacusta* from each sampled population were carefully cleansed and, to eliminate residual ethanol, soaked in double-distilled water for 5 min. From these, total genomic DNA was then extracted using BIORON GmbH “Ron’s Tissue DNA Mini Kit”, following the manufacturer’s instructions. Polymerase chain reactions (PCRs) were then performed to amplify the target mtDNA COI sequence using the primer pair “dgLCO1490” and “dgHCO2198” [35].

The PCR mix consisted of 18.7 μL of distilled water, 2.5 μL of Buffer 10×, which includes 15 mM of MgCl_2_, 0.5 μL of dNTPs (10 mM for each), 0.5 μL of each of the primers (10 μM), 0.3 μL of Taq polymerase (5 U/μL), and 2 μL of template DNA, for a total volume of 25 μL. The thermal cycle consisted of 38 cycles of denaturation (94 °C for 3 min), annealing (48 °C for 45 s), and extension (72 °C for 45 s), followed by 5 min at 72 °C for the final extension step. Subsequently, gel electrophoresis was performed, for each PCR product (volume = 5 µL), on 2% agarose gel at 90 V for 20 min. Electrophoretic plates were then inspected with a UV transilluminator to verify the presence of target sequences. Samples that showed a clear single band of the expected length were then purified using the Exo-SAP-IT^®^ kit (Affymetrix USB, Santa Clara, CA, USA). Sequencing was later performed on the purified samples at the Macrogen Spain Laboratory (https://dna.macrogen.com/eng/) using an ABI 3130xL (Applied Biosystems, Waltham, MA, USA) sequencer. The primers used previously for PCR were later used for direct sequencing of the PCR products.

To recognize new sequences, chromatograms were analyzed and proofread manually using the software Chromas v. 2.6.2 (Technelysium, Pty. Ltd., South Brisbane, Australia). In addition, to compare novel sequences with those already published, 32 *Craspedacusta sowerbii* s.l. sequences and 1 *Maeotias marginata* (Modeer, 1791) (used as an outgroup) sequence were downloaded from GenBank (see Table 1 for their Accession Numbers, A.N.). Sequences were aligned with MEGA11 [36] using the ClustalW method. Error check was performed by visual inspection to identify potential misalignments and by amino acid conversion to identify frameshifts and stop codons, which would indicate the presence of sequencing errors or pseudogenes. To align the novel sequences of the mtDNA COI fragments (length = 591 bp), the relatively short eDNA fragments (=262–287 bp) obtained by eDNA analyses, and those downloaded from GenBank, we trimmed out the tails of the sequences that were absent in some mtDNA COI fragment, thus obtaining a final alignment of 235 bp (“complete dataset”, see Table S1). However, in order not to lose phylogenetic information, we also produced a second dataset including only the longer COI sequences, thus obtaining a 564 bp long alignment (“partial dataset”, see Table S2).

Bayesian inference of phylogeny (BI), as implemented in MrBayes v. 3.2.7 [37], and maximum likelihood (ML) analyses, using PhyML v. 3.0 [38], were performed on both the “complete” and “partial” datasets to investigate the phylogenetic relationships among the sequences. Model selection was performed using the following criteria: nst = mixed, rates = gamma. Node supports were evaluated by their posterior probabilities in the BI, and by 1000 bootstrap replicates in the ML analyses implementing a GTR+G+I evolutionary model. The BI analysis consisted of two independent Markov Chain Monte Carlo simulations performed with the following parameters: generations = 10^6^; temp. = 0.2; priors = default. The trees and parameter values were sampled every 100 generations, resulting in 10,000 trees for each analysis.

The convergence in the analysis was reached (Effective Sample Size (ESS) greater than 473.22 for the “complete” and 558.38 for the “partial” dataset). The first 25% of trees were discarded as “burn-in” in both analyses.

All the available Italian *Craspedacusta sowerbii* mtDNA COI sequences were also used to build a median-joining network based on the “complete” dataset through the software PopART v. 1.7 (http://popart.otago.ac.nz) following Bandelt et al. [39].

### 2.3. Environmental DNA

Pelagic waters, and littoral biofilm of lakes and rivers, were sampled for environmental DNA analysis. Sampling locations and methods were described in Salmaso et al. [40]; Supplementary Tables S1 and S2; and Domaizon et al. [32]. Sampling of Lake Albano was conducted by ARPA Lazio as part of the Eco-AlpsWater activities [33].

Lake water samples were collected from the deepest points of epilimnetic or euphotic zones, then filtered (within 12 h) with Sterivex cartridges (0.22 μm, Hydrophilic PVDF Durapore membrane, Sigma Aldrich, St. Louis, Missouri, USA), and immediately frozen at −20 °C. Lake biofilm samples were collected mostly from September to October, whereas river biofilm samples were collected from February to October by brushing the surface of at least 5 stones following Rimet et al. [41,42].

DNA was extracted, respectively, from Sterivex filters and directly from biofilm, with a Mo Bio PowerWater^®^ DNA Isolation Kit (MO BIO Laboratories, QIAGEN, Venlo, Netherlands) and a NucleoSpin^®^ Soil kit (Macherey-Nagel, Düren, Deutschland) [43,44]. The final DNA library was sequenced on an Illumina^®^ MiSeq (PE300) platform. Original sequences have been deposited in the European Nucleotide Archive (ENA) (study accession number PRJEB49184), and a bioinformatic workflow applied to raw reads, as described in Salmaso et al. [45]. Additional analyses of mtDNA COI sequences were performed on two samples collected from the biofilm of Garda Lake (Lazise) and two samples collected from the biofilm and surface water of Albano Lake using the same sampling protocols described above. Amplification of the COI marker was performed using the mlCOIintF and jgHCO2198 primers [46,47]. Raw reads were deposited to the ENA repository with project number PRJEB70388, and analyzed using the DADA2 protocols described in Callahan et al. [48]. Sequences from Lake Maggiore were obtained from a COI metabarcoding project to monitor zooplankton in the lake [34].

## 3. Results

### 3.1. Distribution of Craspedacusta Sightings in Italy

**Figure 1:**
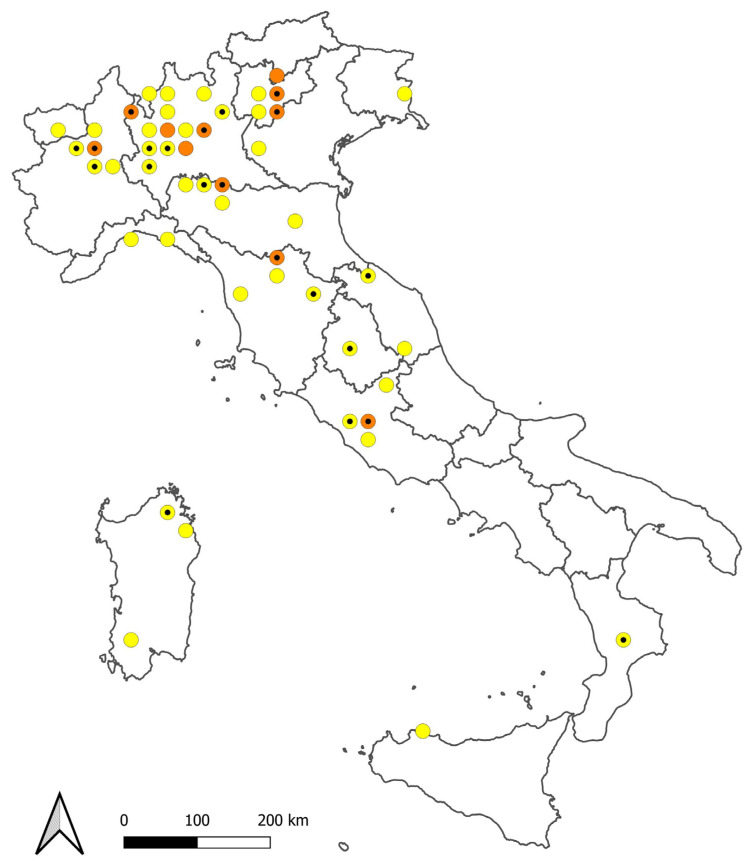
Sightings of *Craspedacusta* grouped on UTM 25 km × 25 km grids. Yellow dots = one site, orange dots = two sites, small black dots = first sighting in the grid made before year 2000.

Overall, the occurrence of *Craspedacusta* was recorded in 61 Italian freshwater habitats (Figure 1 and Table 2). Most records refer to the presence of the pelagic medusae. In Small Lake Monticolo, only polyps were found. Environmental DNA surveys confirmed earlier findings of the jellyfish in Lake Garda and Lake Maggiore and revealed its presence in two previously unreported sites: Lake Albano (mtDNA COI) and River Adige (based on 18S rRNA [40]). In some water bodies, repeated sighting of medusae were reported for several consecutive summers; for example, in Large Lake Monticolo, jellyfish were sighted in eight summers from 2015 to 2023.

The sightings of *Craspedacusta* occurred mainly in Northern Italy (Figure 1), with 90.2% of the sites located below 800 m a.s.l., and the highest site (Lake Arvo) at 1280 m a.s.l. (Table 2).

**Figure 2:**
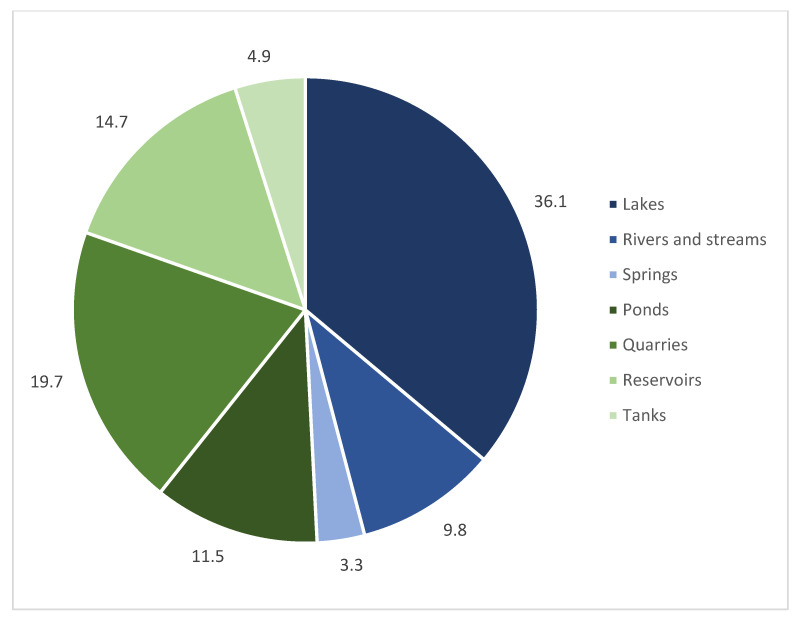
Percentage of *Craspedacusta* sightings by habitat type. Blue shadings represent natural habitat types (lakes, rivers, streams, and springs); green shadings represent artificial habitat types (man-made ponds, water-filled quarries, reservoirs, and tanks).

Sightings of *Craspedacusta* were seen almost equally in natural habitat types (49.2%): large subalpine (i.e., Lake Garda, Lake Maggiore, Lake Ceresio / Lugano, and Lake Como), perialpine (i.e., Lake Levico, Lakes Monticolo), alpine (i.e., Lake Santo Cembra and Lake Lavarone) and volcanic lakes (Lake Albano and Lake Martignano), and rivers (River Adige and River Tiber) and artificial habitat types (50.8%): man-made ponds, reservoirs, and water-filled quarries (Figure 2). Records comprise manly lentic waters and only a few refer instead to lotic habitats (rivers, streams, and springs) (Table 2). Sightings were made generally between July and October when optimal temperature for pelagic jellyfish development is reached.

Gonadal tissue analysis of the specimens shows that the medusae collected in October 2022 in Lake Levico (n = 2) were both females, while the medusae sampled in September 2023 in Lake Santo of Monte Terlago (n = 17) were 16 females and 1 male.

### 3.2. Molecular Analyses

**Figure 3:**
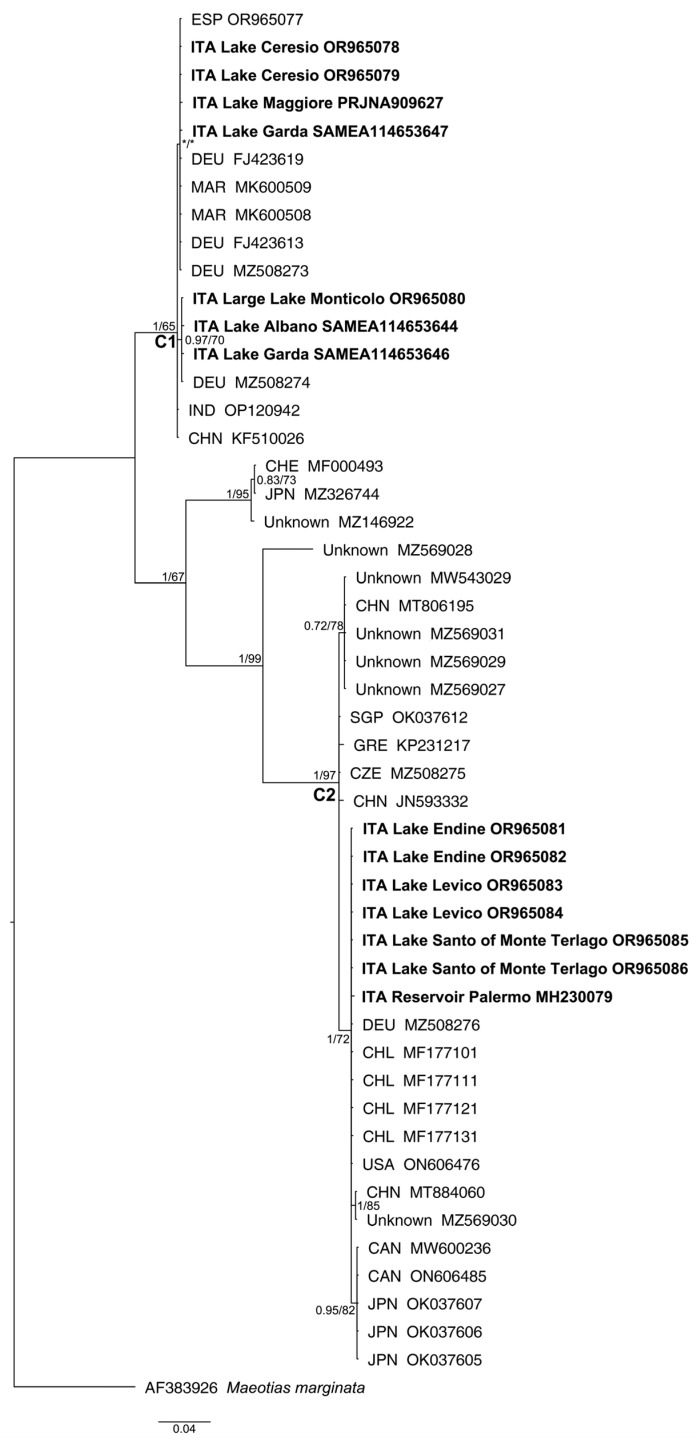
Bayesian phylogram of published and collected *Craspedacusta* specimens based on the mtDNA COI “complete” dataset. *Maeotias marginata* was used as an outgroup to root the tree. Node statistical support is reported as nodal posterior probabilities (Bayesian inference of phylogeny, BI)/bootstrap values (maximum likelihood, ML). Asterisks show support values lower than 50. Italian sequences are reported in bold. CAN, Canada; CHE, Switzerland; CHL, Chile; CHN, China; CZE, Czech Republic; DEU, Germany; ESP, Spain; GRE, Greece; IND, India; ITA, Italy; JPN, Japan; MAR, Morocco; SGP, Singapore.

Overall, we produced nine novel *Craspedacusta* mtDNA COI sequences from Italy and one from Spain. We analyzed 10 specimens of *Craspedacusta* medusae collected from seven water bodies and three sequences of eDNA sampled from other three lakes (see Table 1 and Figure 3). Furthermore, two different COI oligotypes were detected in the four samples collected in Lakes Garda and Albano, two in Lake Garda and one, common in both lakes, in Lake Albano. Results of the mtDNA COI translation into amino acids did not reveal stop codons and showed an amino acid configuration shared across the sequences.

**Figure 4:**
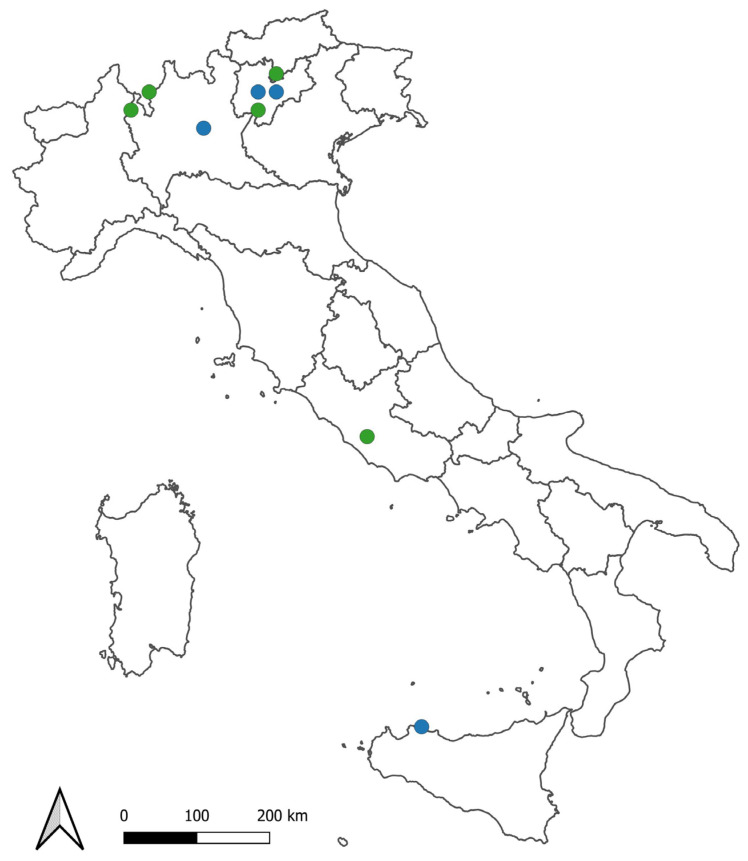
Presence of genetic lineages of *Craspedacusta* in Italy grouped on UTM 25 km × 25 km grids. Green dots = lineage “C1”, blue dots = lineage “C2”.

**Figure 5:**
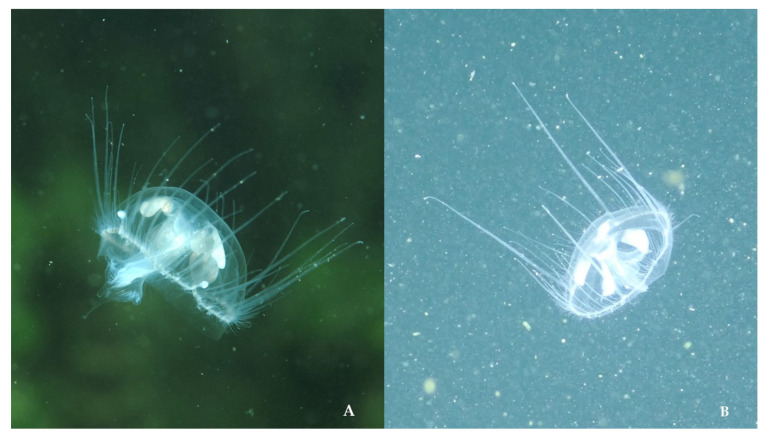
Underwater photos of *Craspedacusta sowerbii* from Italy: (**A**) medusa in Large Lake Monticolo belonging to “clade C1” (photo by Massimo Morpurgo, 30 June 2017); (**B**) medusa in Lake Santo of Monte Terlago belonging to “clade C2” (photo by Kristian Segnana, 20 September 2023).

Bayesian inference of phylogeny (BI) and maximum likelihood (ML) trees based on the mtDNA “complete” COI dataset showed a congruent topology (Figure 3) in agreement with other studies [13,20]. The same tree topology was obtained based on the “partial” dataset (Figure S1). The mtDNA COI fragments of *C. sowerbii* s.l. sequenced in our study clustered in two distinctive genetic lineages (uncorrected “*p*”-distances = 15.7%): (i) clade C1, which comprises sequences whose geographical distribution ranges from central to northern Italy (Figure 4); (ii) clade C2, which comprises three populations from northern Italy, i.e., those from Lake Levico, Lake Santo of Monte Terlago (Figure 5), and Lake Endine, and the single known Sicilian population. In the present study, clade “C1” corresponds to the clade reported as “*C. sowerbii*” by Schifani et al. [20] and to the “*C. sowerbii* type 1” by Schachtl [12] and Wang [14]; conversely, “clade C2” corresponds to the “*C. sowerbii* type 2” by Schachtl [12] and Wang [14].

**Figure 6:**
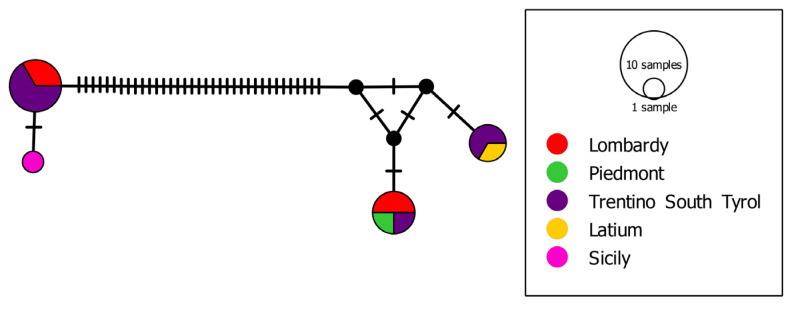
Median-joining haplotype network based on Italian *Craspedacusta* sequences from the mtDNA COI “complete” dataset. Dashes show substitution steps. Each circle represents a haplotype, and its size is proportional to its frequency.

The haplotype network, in agreement with the phylogenetic trees, showed two distinct phylogroups distanced by 35 mutational steps (see Figure 6).

The single Spanish *Craspedacusta* specimen clustered within the C1 lineage.

## 4. Discussion

### 4.1. Distribution of Craspedacusta Sightings in Italy

The genus *Craspedacusta* comprises freshwater jellyfishes that are amongst the most widespread non-native species in inland waters. These inhabit different habitat types (e.g., ponds, lakes, rivers), and their preference for artificial or natural habitats is still under debate [79]. Although we mostly found the species in large subalpine lakes and large artificial reservoirs, according to Marchessaux et al. [7], small-sized habitats, such as water-filled quarries, small lakes, ponds, tanks, seem to facilitate its development.

In Italy, its presence has been revealed in 61 sites; the sightings (and in some cases the collections) generally refer to pelagic medusae. However, eDNA surveys can also reveal the presence of the polyp stage [33,34], and can play a key role in providing a more realistic knowledge of the species’ distribution [15,40].

The present study provides an overview of the presence of the *Craspedacusta* freshwater jellyfish in Italy and contributes to the knowledge of its presence in Europe by complementing the works carried out in Germany [12,14,17,80], France [7], and Spain and Portugal [81], providing also the first molecular data for from Spain (see Figure 3—A.N. OR965077).

Frequently, sightings of medusae are idiosyncratic; for example, made by amateur scuba divers, tourists, and anglers in quarries, lakes, or ponds, and we do not have sound information about the environmental features of the habitats in which sightings are made since these are not often included in environmental studies or monitoring programs. Sightings of *Craspedacusta* increased considerably in the last ten years. However, this trend could either indicate an effective increase of the species’ distribution, or an increased observational effort [23] and faster exchange of photos and videos via social media, through platforms such as iNaturalist and YouTube.

Outside China, unisexual populations of *Craspedacusta* medusae are often observed, and their sexual reproduction seems infrequent [82,83]. Little information is reported in the literature on the gender of freshwater jellyfish populations in Italy because the gonads were analyzed in only a few studies, and in some of these the gonads were not yet mature [26,53,66]. In Large Lake Monticolo, only males were found (n = 30) [13]. In a lake near Schienti, the two medusae analyzed were found to be both females (n = 2) [61], as were the two medusae we sampled in October 2022 in Lake Levico (n = 2). In these two cases, as the sample is too small, the presence of the other gender cannot be ruled out. In Lake Viverone in 1966, both genders were identified: 2 male, 10 female, and 8 unidentified individuals from a sample of 20 medusae [53]. Thus, our finding in September 2023 in Lake Santo of Monte Terlago of 16 females and 1 male (n = 17) represents the second currently known case in Italy of a population with both genders.

### 4.2. Genetic Diversity of Craspedacusta in Italian Inland Waters

The analysis of the available *Craspedacusta* mtDNA COI sequences agrees with previous studies [20,21,23,24,25], and it supports the existence of at least three distinctive clades within *Craspedacusta sowerbii* s.l.: “clade C1”, which comprises sequences from Morocco, Spain, Central–Northern Italy, Germany, India, and the Chinese province of Sichuan (Figure 3); “clade C2”, which comprises sequencies from Chile, Canada, the USA, Sicily (Southern Italy), Northern Italy, Germany, Czech Republic, Greece, China, Singapore, and Japan, and six sequences of unknown origin; and “Clade C3”, which comprises a sequence from Switzerland (MF000493 Ringwiler Weier), one from Japan (MZ326744 Osaka), and one of unknown origin. A further clade, although still to be verified, could comprise a sequence of unknown origin (A.N., MZ569028). The unstable placement of the third clade with the two identical Swiss (Ringwiler Weier) and Japanese (Osaka) mtDNA COI sequences have already been highlighted [21,23,24,25] and needs more comprehensive molecular sampling from these and other locations around the world to be clarified [25]. Our mtDNA COI analysis supports for uncorrected p-distances between clades “C1” and “C2” at 15.7%, in agreement with values found in other studies: 15% [19] and 14% [12].

Our analysis of mtDNA COI confirms the presence in Italy of two genetic lineages of *Craspedacusta sowerbii* s.l. already reported by Morpurgo et al. [13] based on the analysis of 16S markers. Moreover, for the first time, we report the presence of the clade “C2” in peninsular Italy (i.e., in the Lake Endine, Lake Santo of Monte Terlago, and Lake Levico); in Italy, clade C2 was so far reported only for Sicily. Unfortunately, no molecular data are currently available for Sardinian *Craspedacusta* populations. Further molecular analyses of known populations are required to increase our knowledge and understanding of the Italian distribution pattern of the clades singled out within the genus *Craspedacusta*.

In Northern Italy, clades C1 and C2 were found in nearby sites. For example: clade “C1” was found in Lake Garda and Large Lake Monticolo, respectively, 37 and 45 km away from Lake Levico, in which instead clade “C2” was found; clade “C2” was also found in Lake Santo of Monte Terlago, which is located about 34 km away from Lake Garda. Peterson et al. [24] reported the occurrence of two lineages of *Craspedacusta sowerbii* in Japan: one in Nagano and the second in Osaka with a relative proximity of the two collection sites (417 km). In Germany, in a large study with sampling of medusae and polyps in numerous lakes, two distinct genetic lineages of *Craspedacusta sowerbii* (*C. sowerbii* type 1 and *C. sowerbii* type 2, corresponding to our “clade C1” and “clade C2”, respectively) with two haplotypes each were identified [12,14]. Medusae belonging to these two different genetic lineages were even collected in the same water body, Neuer Baarer Weiher, and in two adjacent lakes near Reichertshofen [12].

For more than a century, it was assumed that only one *Craspedacusta* species has spread throughout Europe [4,12]. In agreement with previous studies, molecular evidence based on mtDNA shows the presence in Italian inland waters of at least two distinct genetic lineages within *Craspedacusta sowerbii* s.l. (clade “C1” and clade “C2”), that presumably represent separate species [12,14,19,20,21,23,24,25]. The nomenclatural solution of the new genetically distinct *Craspedacusta* species remains problematic and a taxonomic revision is needed [12,25]. Genetic analysis of the holotypes and paratypes of distinct species of *Craspedacusta* preserved in zoological collections of museums is mandatory to define which is the true *Craspedacusta sowerbii* [12]. Alternatively, as suggested in other studies [21,25], medusae could be sampled in southern England with the aim of detecting the clade corresponding to *Craspedacusta sowerbii* described by Lankester [1].

## 5. Conclusions

Based on the available data, the distribution of *Craspedacusta* in Italy is not yet exhaustively known. Since almost all the records are based on the chance observation of medusae, which make a random appearance, the distribution described in this work is certainly underestimated, and the apparent increase in the number of records in recent decades is probably related to the greater ease with which news and photos are shared via the Internet than in the past. There is a lack of data to ascertain whether the local distribution of the genus *Craspedacusta* is currently expanding, contracting, or stable in recent years in Italy. Although possibly representing different species, the two *Craspedacusta* clades found in Italy do not seem to be characterized by different ecological *preferenda*, and their distributions are patchy and widely overlapping. Further accurate ecological analyses are desirable to check whether some ecological factors overlooked in the frame of the current large-scale survey might provide a better characterization of the autoecologies of these clades. A wide application of eDNA studies would be able to give us standardized information on the presence of the genus, as it can be detected even when present only as polyps [15,16]. In addition, environmental DNA analysis could also allow the investigation of the distribution of the two distinct genetic lineages, quite likely two different species [12,14,19,20,21,23,24,25], which could perhaps even be found in the same water bodies as recorded in Germany [12].

## Figures and Tables

**Table 1:** List of Italian and Spanish sampling sites for which molecular data (mtDNA COI) are available. GenBank Accession Numbers or BioProject/Biosample codes are reported.

Water Body	Sampling Date	Type of Sample	Accession Numbers/BioProject Number/BioSample Codes	Source
Lake Ceresio/Lugano	31 August 2013	1 medusa	OR965078	Present Study
Lake Ceresio/Lugano	13 September 2013	1 medusa	OR965079	Present Study
Concrete reservoir (University of Palermo)	14 November 2017	1 medusa	MH230079	[13]
Large Lake Monticolo	22 July 2018	1 medusa	OR965080	Present Study
Lake Garda	19 April 2021	eDNA	SAMEA114653646–SAMEA114653647	[32]
Lake Albano	9 June 2021	eDNA	SAMEA114653644	[33]
Lake Maggiore	1 August 2021	eDNA	PRJNA909627	[34]
Lake Endine	29 August 2022	2 medusae	OR965081–OR965082	Present Study
Lake Levico	8 October 2022	2 medusae	OR965083–OR965084	Present Study
Lake Santo of Monte Terlago	24 September 2023	2 medusae	OR965085–OR965086	Present Study
Canelles Reservoir	8 October 2019	1 medusa	OR965077	Present Study

**Table 2:** Overview of the sightings of the jellyfish *Craspedacusta* in Italy in chronological order. Legend: * Water bodies with multiple sightings of jellyfish, ** sightings of jellyfish blooms, *** records based on eDNA, **** recorded as polyp stage. Origin: n = natural, a = artificial; Source: sa = scientific article, psa = popular science article, pc = personal communication, w = website, pa = press article.

ID	Year	Water Body Name	Latitude	Longitude	Altitude m a.s.l.	Origin	Habitat	Source	Type of Source
1	1946	Tank (University of Roma)	41.90633	12.51538	54	a	tank (aquarium)	[26]	sa
2	1950	Lake Arvo	39.23887	16.50258	1280	a	reservoir	[49]	sa
3	1950	Lake Suviana	44.12549	11.04076	470	a	reservoir	[50]	sa
4	1963	Milan Idroscalo */**	45.46407	9.28908	107	a	reservoir	[51]	psa
5	1965	Peat bog Iseo	45.64028	10.02231	188	a	quarry	[52]	sa
6	1966	Lake Viverone	45.41590	8.03541	230	n	lake	[53]	psa
7	1969	Lake Sirio *	45.48659	7.88401	266	n	lake	[53,54]	psa, sa
8	1970	Lake Liscia **	41.00075	9.26038	177	a	reservoir	[55]	sa
9	1970	Lake Nero *	45.50514	7.87432	299	n	lake	[53]	psa
10	1970	Lake Maggiore */***	45.97500	8.65250	193	n	lake	[34,56]	sa
11	1972	River Po	45.09305	9.90473	42	n	river (bight)	[57]	sa
12	1974	River Tevere	42.97612	12.40782	167	n	river	[52]	sa
13	1978	Spring (near Casalmaggiore) *	44.99152	10.40448	23	n	spring	[58]	psa
14	1983	Lake near Bibbiena	43.72033	11.83523	425	a	pond	[59]	psa
15	1985	Lake Monate */**	45.79546	8.66476	266	n	lake	[60]	psa
16	1987	Lake near Assago	45.39904	9.10919	109	a	quarry	[57]	sa
17	1988	River Ticino (Bight Topo) **	45.19014	9.11958	77	n	river (bight)	[57]	sa
18	1991	Lake near Schienti */**	43.78850	12.63709	130	a	quarry	[61]	sa
19	1992	Lake Santo Cembra	46.19595	11.20814	1194	n	lake	[28]	sa
20	1995	Lake Lavarone	45.93667	11.25274	1100	n	lake	[28]	sa
21	1996	Lake Moro	45.88095	10.15841	381	n	lake	[62]	w
22	1997	Lake Martignano	42.11330	12.31488	202	n	lake	Present study [Seminara M., *pers. comm.*, 2022]	pc
23	1999	Lake Candia *	45.32467	7.91187	226	n	lake	Present study [Fogliati P., *pers. comm.*, 2022]	pc
24	2002	Lake Svizzera	44.72999	10.20465	146	a	pond	[27]	psa
25	2003	Lake Poiani	45.76825	11.13692	882	a	quarry	[28]	sa
26	2006	Lake Ca’ Stanga	45.05362	9.79581	46	a	quarry	[27]	psa
27	2006	Spring (near Gussola)	45.00539	10.34674	27	n	spring	[27]	psa
28	2006	Lake Alserio	45.78627	9.21383	262	n	lake	[27]	psa
29	2006	Lake Bilancino *	43.97764	11.26552	252	a	reservoir	[63]	sa
30	2007	Lake De Poli (near Rivolta d’Adda) *	45.48849	9.50647	101	a	quarry	[27]	psa
31	2007	Lake Riflessi	45.39773	9.71141	86	a	quarry	[27]	psa
32	2008	Lake Garda */***	45.85534	10.84994	65	n	lake	[32,64]	sa
33	2008	Lake (urban park in Roma)	41.91477	12.48296	60	a	pond (urban)	[27]	psa
34	2009	Lake Leni	39.41441	8.71138	252	a	reservoir	Present study [Satta C.T., *pers. comm.*, 2022]	pc
35	2009	Lake Oasi di Baggero	45.77118	9.23735	253	a	quarry	[65]	w
36	2009	Lake Malpaga **	45.77222	9.23222	330	a	reservoir	[66]	sa
37	2010	Lake Quercia	45.58286	8.13508	316	a	pond	[67]	w
38	2011	Lake Cassiana	43.91886	11.19853	165	a	quarry	[68]	w
39	2012	Lake Gerosa	42.89054	13.37569	650	a	reservoir	[69]	w
40	2012	Lake near Pontedera	43.66787	10.57206	12	a	quarry	[68]	w
41	2013	Lake Ceresio/Lugano */**	46.01674	9.07462	271	n	lake	[70], present study [Lepori F., *pers. comm.*, 2022]	psa, pc
42	2015	Lake Levico */**	46.01560	11.27669	440	n	lake	[28], present study [Tabarelli de Fatis K., *pers. comm.*, 2022, Segnana K., *pers. comm.*, 2023]	sa, pc
43	2015	Tank (near Lovoleto)	44.58153	11.43169	22	a	tank (soaking tank)	[71]	sa
44	2015	Lake Como	46.03133	9.26840	198	n	lake	[72]	w
45	2015	River Po	45.14266	8.44953	108	n	river (bight)	[73]	sa
46	2015	Large Lake Monticolo */**	46.42331	11.29004	492	n	lake	[73]	sa
47	2015	Lake Brissogne	45.73762	7.40175	830	a	quarry	[73]	sa
48	2016	Lake Montorfano	45.78276	9.13786	397	n	lake	[28]	sa
49	2017	Concrete reservoir (University of Palermo)	38.10700	13.35086	40	a	tank (holding tank)	[20]	sa
50	2018	Lake (urban park of Arenzano)	44.40178	8.68178	23	a	pond (urban)	[74]	pa
51	2018	Small Lake Monticolo ****	46.42963	11.29611	514	n	lake	[13]	sa
52	2018	Small Lake Bocco	44.41394	9.44867	956	a	reservoir	Present study [Cresta P., *pers. comm.*, 2023]	pc
53	2019	River Adige ***	45.48138	10.82939	77	n	river	[40]	sa
54	2019	Lake (near Campeglio) */**	46.12341	13.37776	145	a	quarry	Present study [Ianesi A., *pers. comm*., 2022]	pc
55	2020	Lake Chiuro	46.16216	10.00398	389	a	pond	[75]	w
56	2021	Lake Albano ***	41.74702	12.67016	293	n	lake	[33]	sa
57	2021	Lake Endine *	45.78129	9.94028	334	n	lake	[76], present study [Pezzini V., *pers. comm.*, 2022]	w, pc
58	2022	Lake Club E-20	45.73264	9.62915	288	a	pond	[77]	w
59	2023	Lake Santo of Monte Terlago	46.12534	11.05934	713	n	lake	Present study	sa
60	2023	Lake Paterno	42.38254	13.01435	430	n	lake	Present study [ Gasparini A., *pers. comm.*, 2023]	pc
61	2023	Stream Fossu Frate di Ghirru, pool Poiu Pitriolu	40.75052	9.59611	290	n	stream pool	[78]	sa

## Data Availability

Raw reads from Illumina sequencing: BioProject PRJNA909627. COI from eDNA, see BioProject PRJEB70388 in methods. GBIF dataset for the occurrence records https://doi.org/10.15468/9t7ead (accessed on 30 November 2023). The data presented in this study are available on GenBank and in the Supplementary Materials.

## Supplementary Materials

The following supporting information can be downloaded at: https://www.mdpi.com/article/10.3390/biology13040202/s1, Figure S1. Bayesian phylogram of published and collected *Craspedacusta* specimens based on the mtDNA COI “partial dataset”. *Maeotias marginata* was used as an outgroup to root the tree. Node statistical support is reported as nodal posterior probabilities (Bayesian inference of phylogeny, BI)/bootstrap values (maximum likelihood, ML). Asterisks show support values lower than 50. Italian sequences are reported in bold. CAN, Canada; CHE, Switzerland; CHN, China; CZE, Czech Republic; DEU, Germany; ESP, Spain; GRE, Greece; IND, India; ITA, Italy; JPN, Japan; MAR, Morocco; SGP, Singapore.; Table S1. *Craspedacusta* mtDNA COI “complete” dataset alignment. Table S2. *Craspedacusta* mtDNA COI “partial” dataset alignment.
