# Supplementary material for: Distribution and Genetic Lineages of the Craspedacusta sowerbii Species Complex (Cnidaria, Olindiidae) in Italy
Source: Biology (Basel). 2024 Mar 22;13(4):202. doi: 10.3390/biology13040202 (PMC11048658; doi:10.3390/biology13040202)
Supplement: Supplementary file 1 [file biology-13-00202-s001.zip › biology-2888692-supplementary.pdf]

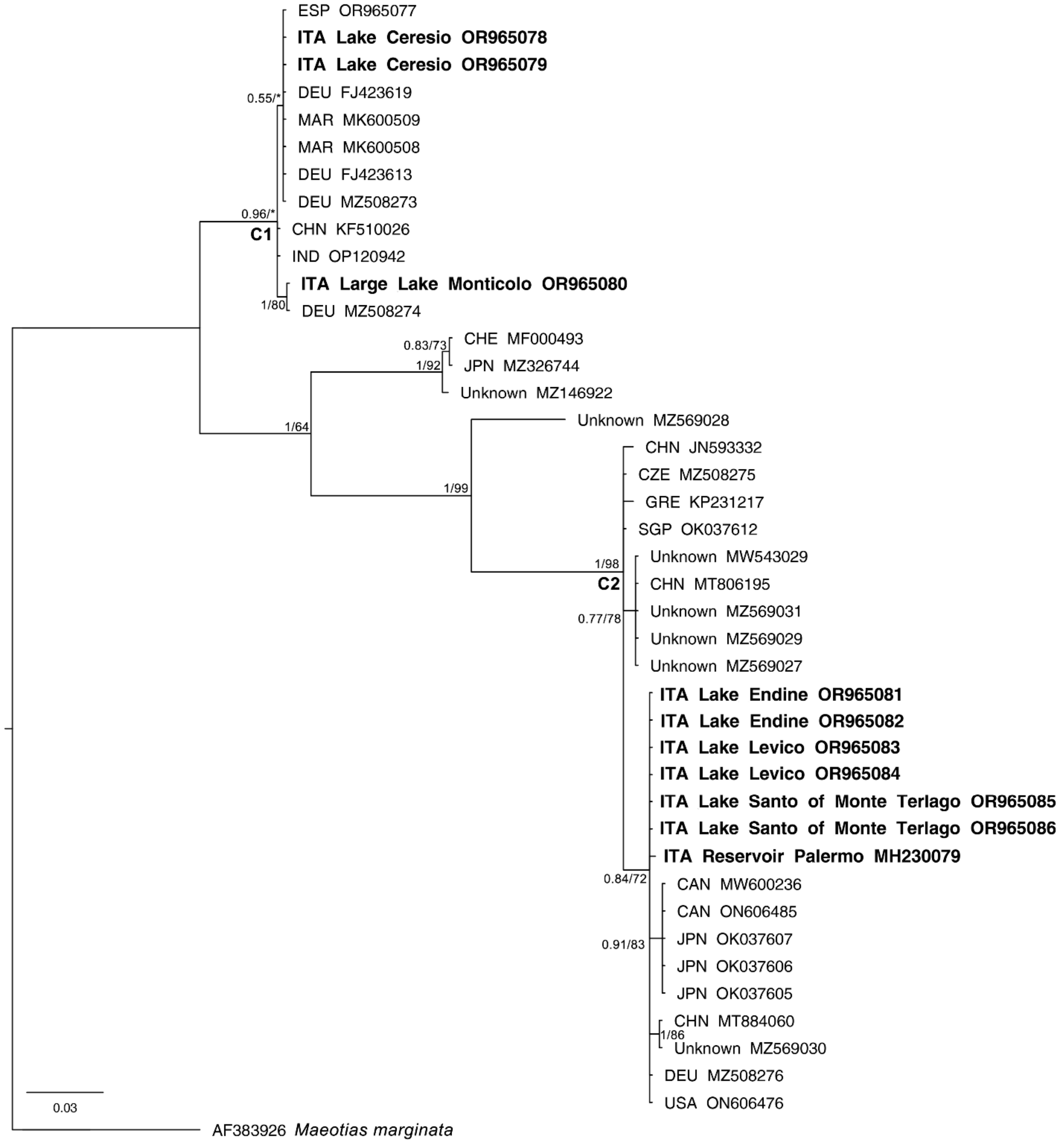

Figure S1: Bayesian phylogram of published and collected *Craspedacusta* specimens based on the mtDNA COI "partial dataset". *Maeotias marginata* was used as an outgroup to root the tree. Node statistical support is reported as nodal posterior probabilities (Bayesian Inference of phylogeny, BI)/bootstrap values (maximum likelihood, ML). Asterisks show support values lower than 50. Italian sequences are reported in bold. CAN, Canada; CHE, Switzerland; CHN, China; CZE, Czech Republic; DEU, Germany; ESP, Spain; GRE, Greece; IND, India; ITA, Italy; JPN, Japan; MAR, Morocco; SGP, Singapore.

Table S1. *Craspedacusta* mtDNA COI “complete” dataset alignment.

>ESP_OR965077<br>GGACCTTCAGTAGACATGGCCATCTTTAGCCTTCATGCGGCAGGTGCTTCCTCCATCATGGGTTCTATGAATTTCATCACTACTATTTCAACATGAGAGCACCAGGAATGA<br>CCATGGATCGCGTTCCCCTCTTCGTTTGAGCTGTACTCATCACGGCATTCCTTTTAGTTCTCTCTTTGCCCGTCTTAGCTGGAGCCATTACTATGTTACTAACTGATCGTAACT<br>TTAATACCA
>ITA_Lake_Ceresio_OR965078<br>GGACCTTCAGTAGACATGGCCATCTTTAGCCTTCATGCGGCAGGTGCTTCCTCCATCATGGGTTCTATGAATTTCATCACTACTATTTCAACATGAGAGCACCAGGAATGA<br>CCATGGATCGCGTTCCCCTCTTCGTTTGAGCTGTACTCATCACGGCATTCCTTTTAGTTCTCTCTTTGCCCGTCTTAGCTGGAGCCATTACTATGTTACTAACTGATCGTAACT<br>TTAATACCA
>ITA_Lake_Ceresio_OR965079<br>GGACCTTCAGTAGACATGGCCATCTTTAGCCTTCATGCGGCAGGTGCTTCCTCCATCATGGGTTCTATGAATTTCATCACTACTATTTCAACATGAGAGCACCAGGAATGA<br>CCATGGATCGCGTTCCCCTCTTCGTTTGAGCTGTACTCATCACGGCATTCCTTTTAGTTCTCTCTTTGCCCGTCTTAGCTGGAGCCATTACTATGTTACTAACTGATCGTAACT<br>TTAATACCA
>ITA_Large_Lake_Monticolo_OR965080<br>GGACCTTCAGTAGACATGGCCATCTTTAGCCTTCATGCGGCAGGTGCTTCCTCCATCATGGGTTCTATGAATTTCATCACTACTATCTTCAACATGAGAGCACCAGGAATGA<br>CCATGGATCGCGTTCCCCTCTTCGTTTGAGCCGTACTCATCACGGCATTCCTTTTAGTTCTCTCTTTGCCCGTCTTAGCTGGAGCGATTACTATGTTACTAACTGATCGTAACT<br>TTAATACCA
>ITA_Lake_Endine_OR965081<br>GGACCTTCCGTGGATATGGCCATCTTTAGCCTTCATGCGGCCGGTGCCTCTTCTATCATGGGATCCATGAACTTCATCACCACCATCTTTAACATGAGAGCCCCAGGGATGA<br>CCATGGATCGCGTTCCTCTCTTCGTCTGGGCAGTGCTAATCACGGCCTTCCTTCTGGTTCTATCTCTTCCCGTCTTAGCAGGAGCCATCACCATGCTACTCACGGACCGTAA<br>CTTCAACACCA
>ITA_Lake_Endine_OR965082<br>GGACCTTCCGTGGATATGGCCATCTTTAGCCTTCATGCGGCCGGTGCCTCTTCTATCATGGGATCCATGAACTTCATCACCACCATCTTTAACATGAGAGCCCCAGGGATGA<br>CCATGGATCGCGTTCCTCTCTTCGTCTGGGCAGTGCTAATCACGGCCTTCCTTCTGGTTCTATCTCTTCCCGTCTTAGCAGGAGCCATCACCATGCTACTCACGGACCGTAA<br>CTTCAACACCA
>ITA_Lake_Levico_OR965083<br>GGACCTTCCGTGGATATGGCCATCTTTAGCCTTCATGCGGCCGGTGCCTCTTCTATCATGGGATCCATGAACTTCATCACCACCATCTTTAACATGAGAGCCCCAGGGATGA<br>CCATGGATCGCGTTCCTCTCTTCGTCTGGGCAGTGCTAATCACGGCCTTCCTTCTGGTTCTATCTCTTCCCGTCTTAGCAGGAGCCATCACCATGCTACTCACGGACCGTAA<br>CTTCAACACCA
>ITA_Lake_Levico_OR965084<br>GGACCTTCCGTGGATATGGCCATCTTTAGCCTTCATGCGGCCGGTGCCTCTTCTATCATGGGATCCATGAACTTCATCACCACCATCTTTAACATGAGAGCCCCAGGGATGA<br>CCATGGATCGCGTTCCTCTCTTCGTCTGGGCAGTGCTAATCACGGCCTTCCTTCTGGTTCTATCTCTTCCCGTCTTAGCAGGAGCCATCACCATGCTACTCACGGACCGTAA<br>CTTCAACACCA
>ITA_Lake_Santo_of_Monte_Terlago_OR965085<br>GGACCTTCCGTGGATATGGCCATCTTTAGCCTTCATGCGGCCGGTGCCTCTTCTATCATGGGATCCATGAACTTCATCACCACCATCTTTAACATGAGAGCCCCAGGGATGA<br>CCATGGATCGCGTTCCTCTCTTCGTCTGGGCAGTGCTAATCACGGCCTTCCTTCTGGTTCTATCTCTTCCCGTCTTAGCAGGAGCCATCACCATGCTACTCACGGACCGTAA<br>CTTCAACACCA
>ITA_Lake_Santo_of_Monte_Terlago_OR965086<br>GGACCTTCCGTGGATATGGCCATCTTTAGCCTTCATGCGGCCGGTGCCTCTTCTATCATGGGATCCATGAACTTCATCACCACCATCTTTAACATGAGAGCCCCAGGGATGA<br>CCATGGATCGCGTTCCTCTCTTCGTCTGGGCAGTGCTAATCACGGCCTTCCTTCTGGTTCTATCTCTTCCCGTCTTAGCAGGAGCCATCACCATGCTACTCACGGACCGTAA<br>CTTCAACACCA
>ITA_Lake_Albano_SAMEA114653644<br>GGACCTTCAGTAGACATGGCCATCTTTAGCCTTCATGCGGCAGGTGCTTCCTCCATCATGGGTTCTATGAATTTCATCACTACTATCTTCAACATGAGAGCACCAGGAATGA<br>CCATGGATCGCGTTCCCCTCTTCGTTTGAGCCGTACTCATCACGGCATTCCTTTTAGTTCTCTCTTTGCCCGTCTTAGCTGGAGCGATTACTATGTTACTAACTGATCGTAACT<br>TTAATACCA
>ITA_Lake_Garda_SAMEA114653646<br>GGACCTTCAGTAGACATGGCCATCTTTAGCCTTCATGCGGCAGGTGCTTCCTCCATCATGGGTTCTATGAATTTCATCACTACTATCTTCAACATGAGAGCACCAGGAATGA<br>CCATGGATCGCGTTCCCCTCTTCGTTTGAGCCGTACTCATCACGGCATTCCTTTTAGTTCTCTCTTTGCCCGTCTTAGCTGGAGCGATTACTATGTTACTAACTGATCGTAACT<br>TTAATACCA
>ITA_Lake_Garda_SAMEA114653647<br>GGACCTTCAGTAGACATGGCCATCTTTAGCCTTCATGCGGCAGGTGCTTCCTCCATCATGGGTTCTATGAATTTCATCACTACTATTTCAACATGAGAGCACCAGGAATGA<br>CCATGGATCGCGTTCCCCTCTTCGTTTGAGCTGTACTCATCACGGCATTCCTTTTAGTTCTCTCTTTGCCCGTCTTAGCTGGAGCCATTACTATGTTACTAACTGATCGTAACT<br>TTAATACCA
>ITA_Lake_Maggiore_PRJNA909627<br>GGACCTTCAGTAGACATGGCCATCTTTAGCCTTCATGCGGCAGGTGCTTCCTCCATCATGGGTTCTATGAATTTCATCACTACTATTTCAACATGAGAGCACCAGGAATGA<br>CCATGGATCGCGTTCCCCTCTTCGTTTGAGCTGTACTCATCACGGCATTCCTTTTAGTTCTCTCTTTGCCCGTCTTAGCTGGAGCCATTACTATGTTACTAACTGATCGTAACT<br>TTAATACCA
>ITA_Reservoir_Palermo_MH230079<br>GGACCTTCCGTGGATATGGTCATCTTTAGCCTTCATGCGGCCGGTGCCTCTTCTATCATGGGATCCATGAACTTCATCACCACCATCTTTAACATGAGAGCCCCAGGGATGA<br>CCATGGATCGCGTTCCTCTCTTCGTCTGGGCAGTGCTAATCACGGCCTTCCTTCTGGTTCTATCTCTTCCCGTCTTAGCAGGAGCCATCACCATGCTACTCACGGACCGTAA<br>CTTCAACACCA
>DEU_MZ508274<br>GGACCTTCAGTAGACATGGCCATCTTTAGCCTTCATGCGGCAGGTGCTTCCTCCATCATGGGTTCTATGAATTTCATCACTACTATCTTCAACATGAGAGCACCAGGAATGA<br>CCATGGATCGCGTTCCCCTCTTCGTTTGAGCCGTACTCATCACGGCATTCCTTTTAGTTCTCTCTTTGCCCGTCTTAGCTGGAGCGATTACTATGTTACTAACTGATCGTAACT<br>TTAATACCA
>IND_OP120942<br>GGACCTTCAGTAGACATGGCCATCTTTAGCCTTCATGCGGCAGGTGCTTCCTCCATCATGGGTTCTATGAATTTCATCACTACTATCTTCAACATGAGAGCACCAGGAATGA<br>CCATGGATCGCGTTCCCCTCTTCGTTTGAGCTGTACTCATCACGGCATTCCTTTTAGTTCTCTCTTTGCCCGTCTTAGCTGGAGCCATTACTATGTTACTAACTGATCGTAACT<br>TTAATACCA
>DEU_FJ423619<br>GGACCTTCAGTAGACATGGCCATCTTTAGCCTTCATGCGGCAGGTGCTTCCTCCATCATGGGTTCTATGAATTTCATCACTACTATTTCAACATGAGAGCACCAGGAATGA<br>CCATGGATCGCGTTCCCCTCTTCGTTTGAGCTGTACTCATCACGGCATTCCTTTTAGTTCTCTCTTTGCCCGTCTTAGCTGGAGCCATTACTATGTTACTAACTGATCGTAACT<br>TTAATACCA
>MAR_MK600509<br>GGACCTTCAGTAGACATGGCCATCTTTAGCCTTCATGCGGCAGGTGCTTCCTCCATCATGGGTTCTATGAATTTCATCACTACTATTTCAACATGAGAGCACCAGGAATGA<br>CCATGGATCGCGTTCCCCTCTTCGTTTGAGCTGTACTCATCACGGCATTCCTTTTAGTTCTCTCTTTGCCCGTCTTAGCTGGAGCCATTACTATGTTACTAACTGATCGTAACT<br>TTAATACCA
>MAR_MK600508<br>GGACCTTCAGTAGACATGGCCATCTTTAGCCTTCATGCGGCAGGTGCTTCCTCCATCATGGGTTCTATGAATTTCATCACTACTATTTCAACATGAGAGCACCAGGAATGA<br>CCATGGATCGCGTTCCCCTCTTCGTTTGAGCTGTACTCATCACGGCATTCCTTTTAGTTCTCTCTTTGCCCGTCTTAGCTGGAGCCATTACTATGTTACTAACTGATCGTAACT<br>TTAATACCA
>CHN_KF510026<br>GGACCTTCAGTAGACATGGCCATCTTTAGCCTTCATGCGGCAGGTGCTTCCTCCATCATGGGTTCTATGAATTTCATCACTACTATCTTCAACATGAGAGCACCAGGAATGA

CCATGGATCGCGTTCCCCTCTTCGTTTGAGCTGTACTCATCACGGCATTCCTTTTAGTTCTCTCTTTGCCCGTCTTAGCTGGAGCCATTACTATGTTACTAACTGATCGTAACT<br>TTAATACCA
>CHE_MF000493<br>GGCCCCTCGGTAGACATGGCCATCTTCAGTCTTCATGCGGCAGGTGCTTCCTCGATCATGGGTTCCATGAACTTCATCACCACCATCTTTAACATGAGAGCCCCAGGAATG<br>ACGATGGATCGCGTACCTCTGTTCGTGTGAGCCGTACTCATCACAGCCTTCCTTTTAGTTCTGTCTTTACCCGTTTTAGCTGGAGCCATTACCATGTTGTTGACAGATCGTAA<br>TTTCAATACGA
>Unknown_MW543029<br>GGACCTTCCGTGGATATGGCCATCTTTAGCCTTCATGCGGCCGGTGCCTCCTCTATCATGGGATCCATGAACTTCATCACCACCATCTTTAACATGAGAGCCCCAGGGATG<br>ACCATGGATCGCGTTCCTCTCTTCGTCTGGGCAGTACTAATCACGGCCTTCCTTCTGGTTCTATCTCTCCCCGTCTTAGCAGGGGCCATCACCATGCTACTCACGGACCGTA<br>ACTTCAACACCA
>CAN_MW600236<br>GGACCTTCCGTGGATATGGCCATCTTTAGCCTTCATGCGGCCGGTGCCTCTTCTATCATGGGATCCATGAACTTCATCACCACCATCTTTAACATGAGAGCCCCAGGGATGA<br>CCATGGATCGCGTTCCTCTCTTCGTCTGAGCAGTGCTAATCACGGCCTTCCTTCTGGTTCTATCTCTTCCCGTCTTAGCAGGAGCCATCACCATGCTACTCACGGACCGTAA<br>CTTCAACACCA
>CAN_ON606485<br>GGACCTTCCGTGGATATGGCCATCTTTAGCCTTCATGCGGCCGGTGCCTCTTCTATCATGGGATCCATGAACTTCATCACCACCATCTTTAACATGAGAGCCCCAGGGATGA<br>CCATGGATCGCGTTCCTCTCTTCGTCTGAGCAGTGCTAATCACGGCCTTCCTTCTGGTTCTATCTCTTCCCGTCTTAGCAGGAGCCATCACCATGCTACTCACGGACCGTAA<br>CTTCAACACCA
>SGP_OK037612<br>GGACCTTCCGTGGATATGGCCATCTTTAGCCTTCATGCGGCCGGTGCCTCCTCTATCATGGGATCCATGAACTTCATCACCACCATCTTTAACATGAGAGCCCCAGGGATG<br>ACCATGGATCGCGTTCCTCTCTTCGTCTGGGCAGTACTAATCACGGCCTTCCTTCTGGTTCTATCTCTCCCCGTCTTAGCAGGAGCCATCACCATGCTACTCACGGACCGTA<br>ACTTCAACACCA
>JPN_OK037607<br>GGACCTTCCGTGGATATGGCCATCTTTAGCCTTCATGCGGCCGGTGCCTCTTCTATCATGGGATCCATGAACTTCATCACCACCATCTTTAACATGAGAGCCCCAGGGATGA<br>CCATGGATCGCGTTCCTCTCTTCGTCTGAGCAGTGCTAATCACGGCCTTCCTTCTGGTTCTATCTCTTCCCGTCTTAGCAGGAGCCATCACCATGCTACTCACGGACCGTAA<br>CTTCAACACCA
>CHN_MT806195<br>GGACCTTCCGTGGATATGGCCATCTTTAGCCTTCATGCGGCCGGTGCCTCCTCTATCATGGGATCCATGAACTTCATCACCACCATCTTTAACATGAGAGCCCCAGGGATG<br>ACCATGGATCGCGTTCCTCTCTTCGTCTGGGCAGTACTAATCACGGCCTTCCTTCTGGTTCTATCTCTCCCCGTCTTAGCAGGGGCCATCACCATGCTACTCACGGACCGTA<br>ACTTCAACACCA
>JPN_OK037606<br>GGACCTTCCGTGGATATGGCCATCTTTAGCCTTCATGCGGCCGGTGCCTCTTCTATCATGGGATCCATGAACTTCATCACCACCATCTTTAACATGAGAGCCCCAGGGATGA<br>CCATGGATCGCGTTCCTCTCTTCGTCTGAGCAGTGCTAATCACGGCCTTCCTTCTGGTTCTATCTCTTCCCGTCTTAGCAGGAGCCATCACCATGCTACTCACGGACCGTAA<br>CTTCAACACCA
>JPN_OK037605<br>GGACCTTCCGTGGATATGGCCATCTTTAGCCTTCATGCGGCCGGTGCCTCTTCTATCATGGGATCCATGAACTTCATCACCACCATCTTTAACATGAGAGCCCCAGGGATGA<br>CCATGGATCGCGTTCCTCTCTTCGTCTGAGCAGTGCTAATCACGGCCTTCCTTCTGGTTCTATCTCTTCCCGTCTTAGCAGGAGCCATCACCATGCTACTCACGGACCGTAA<br>CTTCAACACCA
>CHN_MT884060<br>GGACCTTCCGTGGATATGGCCATCTTTAGCCTTCATGCGGCCGGTGCCTCTTCTATCATGGGATCCATGAACTTCATCACCACCATCTTTAACATGAGAGCCCCAGGGATGA<br>CCATGGATCGCGTTCCTCTCTTCGTCTGGGCAGTGCTAATCACGGCCTTCCTTCTGGTTCTATCTCTTCCCGTCTTGGCAGGAGCCATCACCATGCTACTCACGGACCGTAA<br>CTTCAACACCA
>GRE_KP231217<br>GGACCTTCCGTGGATATGGCCATCTTTAGCCTTCATGCGGCCGGTGCCTCCTCTATCATGGGATCCATGAACTTCATCACCACCATCTTTAACATGAGAGCCCCAGGGATG<br>ACCATGGATCGCGTTCCTCTCTTCGTCTGGGCAGTACTAATCACGGCCTTCCTTCTGGTTCTATCTCTCCCCGTCTTAGCAGGAGCCATCACCATACTACTCACGGACCGTA<br>ACTTCAACACCA
>DEU_FJ423613<br>GGACCTTCAGTAGACATGGCCATCTTTAGCCTTCATGCGGCAGGTGCTTCCTCCATCATGGGTTCTATGAATTTCATCACTACTATTTTCAACATGAGAGCACCAGGAATGA<br>CCATGGATCGCGTTCCCCTCTTCGTTTGAGCTGTACTCATCACGGCATTCCTTTTAGTTCTCTCTTTGCCCGTCTTAGCTGGAGCCATTACTATGTTACTAACTGATCGTAACT<br>TTAATACCA
>JPN_MZ326744<br>GGCCCCTCGGTAGACATGGCCATCTTCAGTCTTCATGCGGCAGGTGCTTCCTCGATCATGGGTTCCATGAACTTCATCACCACCATCTTTAACATGAGAGCCCCAGGAATG<br>ACGATGGATCGCGTACCTCTGTTCGTGTGAGCCGTACTCATCACAGCCTTCCTTTTAGTTCTGTCTTTACCCGTTTTAGCTGGAGCCATTACCATGTTGTTGACAGATCGTAA<br>TTTCAATACGA
>Unknown_MZ146922<br>GGCCCCTCGGTAGACATGGCCATCTTCAGTCTTCATGCGGCAGGTGCTTCCTCGATCATGGGTTCCATGAACTTCATCACCACCATCTTTAACATGAGAGCCCCAGGAATG<br>ACGATGGATCGCGTACCTCTGTTCGTGTGAGCCGTACTCATCACAGCCTTCCTTTTAGTTCTGTCTTTACCCGTTTTAGCTGGAGCCATTACCATGTTGTTGACAGATCGTAA<br>TTTCAATACGA
>Unknown_MZ569028<br>GGGCCTTCGGTAGACATGGCCATCTTCAGCCTTCACGCGGCTGGTGCCTCCTCTATCATGGGATCCATGAATTTCATCACCACCATCTTTAACATGAGAGCCCCAGGGATG<br>ACGATGGATCGAGTCCCTCTCTTCGTTTGAGCAGTGCTAATCACGGCCTTTCTGTTAGTCCTCTCTCTACCCGTTTTAGCGGGGGCCATCACCATGTTGCTCACGGATCGTA<br>ATTTCAACACCA
>Unknown_MZ569031<br>GGACCTTCCGTGGATATGGCCATCTTTAGCCTTCATGCGGCCGGTGCCTCCTCTATCATGGGATCCATGAACTTCATCACCACCATCTTTAACATGAGAGCCCCAGGGATG<br>ACCATGGATCGCGTTCCTCTCTTCGTCTGGGCAGTACTAATCACGGCCTTCCTTCTGGTTCTATCTCTCCCCGTCTTAGCAGGGGCCATCACCATGCTACTCACGGACCGTA<br>ACTTCAACACCA
>Unknown_MZ569029<br>GGACCTTCCGTGGATATGGCCATCTTTAGCCTTCATGCGGCCGGTGCCTCCTCTATCATGGGATCCATGAACTTCATCACCACCATCTTTAACATGAGAGCCCCAGGGATG<br>ACCATGGATCGCGTTCCTCTCTTCGTCTGGGCAGTACTAATCACGGCCTTCCTTCTGGTTCTATCTCTCCCCGTCTTAGCAGGGGCCATCACCATGCTACTCACGGACCGTA<br>ACTTCAACACCA
>Unknown_MZ569027<br>GGACCTTCCGTGGATATGGCCATCTTTAGCCTTCATGCGGCCGGTGCCTCCTCTATCATGGGATCCATGAACTTCATCACCACCATCTTTAACATGAGAGCCCCAGGGATG<br>ACCATGGATCGCGTTCCTCTCTTCGTCTGGGCAGTACTAATCACGGCCTTCCTTCTGGTTCTATCTCTCCCCGTCTTAGCAGGGGCCATCACCATGCTACTCACGGACCGTA<br>ACTTCAACACCA
>CZE_MZ508275<br>GGACCTTCCGTGGATATGGCCATCTTTAGCCTTCATGCGGCCGGTGCCTCCTCTATCATGGGATCCATGAACTTCATCACCACCATCTTTAACATGAGAGCCCCAGGGATG<br>ACCATGGATCGCGTTCCTCTCTTCGTCTGGGCAGTACTAATCACGGCCTTCCTTCTGGTTCTATCTCTCCCCGTCTTAGCAGGAGCCATCACCATGCTACTCACGGACCGTA<br>ACTTCAACACCA
>CHN_JN593332<br>GGACCTTCCGTGGATATGGCCATCTTTAGCCTTCATGCGGCCGGTGCTTCCTCTATCATGGGATCCATGAACTTCATCACCACCATCTTTAACATGAGAGCCCCAGGGATGG<br>CCATGGATCGCGTTCCTCTCTTCGTCTGGGCAGTACTAATCACGGCCTTCCTTCTGGTTCTATCTCTCCCCGTCTTAGCAGGAGCCATCACCATGCTACTCACGGACCGTAA<br>CTTCAACACCA
>DEU_MZ508276<br>GGACCTTCCGTGGATATGGCCATCTTTAGCCTTCATGCGGCCGGTGCCTCTTCTATCATGGGATCCATGAACTTCATCACCACCATCTTTAACATGAGAGCCCCAGGGATGA<br>CCATGGATCGCGTTCCTCTCTTCGTCTGGGCAGTGCTAATCACGGCCTTCCTTCTGGTTCTATCTCTTCCCGTCTTAGCAGGAGCCATCACCATGCTACTCACGGACCGTAA<br>CTTCAACACCA

>Unknown_MZ569030<br>GGACCTTCCGTGGATATGGCCATCTTTAGCCTTCATGCGGCCGGTGCCTCTTCTATCATGGGATCCATGAACTTCATCACCACCATCTTTAACATGAGAGCCCCAGGGATGA<br>CCATGGATCGCGTTCCTCTCTTCGTCTGGGCAGTGCTAATCACGGCCTTCCTTCTGGTTCTATCTCTTCCCGTCTTGGCAGGAGCCATCACCATGCTACTCACGGACCGTAA<br>CTTCAACACCA
>DEU_MZ508273<br>GGACCTTCAGTAGACATGGCCATCTTTAGCCTTCATGCGGCAGGTGCTTCCTCCATCATGGGTTCTATGAATTTCATCACTACTATTTTCAACATGAGAGCACCAGGAATGA<br>CCATGGATCGCGTTCCCCTCTTCGTTTGAGCTGTACTCATCACGGCATTCCTTTTAGTTCTCTCTTTGCCCGTCTTAGCTGGAGCCATTACTATGTTACTAACTGATCGTAACT<br>TTAATACCA
>CHL_MF177101<br>GGACCTTCCGTGGATATGGCCATCTTTAGCCTTCATGCGGCCGGTGCCTCTTCTATCATGGGATCCATGAACTTCATCACCACCATCTTTAACATGAGAGCCCCAGGGATGA<br>CCATGGATCGCGTTCCTCTCTTCGTCTGGGCAGTGCTAATCACGGCCTTCCTTCTGGTTCTATCTCTTCCCGTCTTAGCAGGAGCCATCACCATGCTACTCACGGACCGTAA<br>CTTCAACACCA
>CHL_MF177111<br>GGACCTTCCGTGGATATGGCCATCTTTAGCCTTCATGCGGCCGGTGCCTCTTCTATCATGGGATCCATGAACTTCATCACCACCATCTTTAACATGAGAGCCCCAGGGATGA<br>CCATGGATCGCGTTCCTCTCTTCGTCTGGGCAGTGCTAATCACGGCCTTCCTTCTGGTTCTATCTCTTCCCGTCTTAGCAGGAGCCATCACCATGCTACTCACGGACCGTAA<br>CTTCAACACCA
>CHL_MF177121<br>GGACCTTCCGTGGATATGGCCATCTTTAGCCTTCATGCGGCCGGTGCCTCTTCTATCATGGGATCCATGAACTTCATCACCACCATCTTTAACATGAGAGCCCCAGGGATGA<br>CCATGGATCGCGTTCCTCTCTTCGTCTGGGCAGTGCTAATCACGGCCTTCCTTCTGGTTCTATCTCTTCCCGTCTTAGCAGGAGCCATCACCATGCTACTCACGGACCGTAA<br>CTTCAACACCA
>CHL_MF177131<br>GGACCTTCCGTGGATATGGCCATCTTTAGCCTTCATGCGGCCGGTGCCTCTTCTATCATGGGATCCATGAACTTCATCACCACCATCTTTAACATGAGAGCCCCAGGGATGA<br>CCATGGATCGCGTTCCTCTCTTCGTCTGGGCAGTGCTAATCACGGCCTTCCTTCTGGTTCTATCTCTTCCCGTCTTAGCAGGAGCCATCACCATGCTACTCACGGACCGTAA<br>CTTCAACACCA
>USA_ON606476<br>GGACCTTCCGTGGATATGGCCATCTTTAGCCTTCATGCGGCCGGTGCCTCTTCTATCATGGGATCCATGAACTTCATCACCACCATCTTTAACATGAGAGCCCCAGGGATGA<br>CCATGGATCGCGTTCCTCTCTTCGTCTGGGCAGTGCTAATCACGGCCTTCCTTCTGGTTCTATCTCTTCCCGTCTTAGCAGGAGCCATCACCATGCTACTCACGGACCGTAA<br>CTTCAACACCA
>AF383926_Maeotias_marginata<br>GGACCTTCTGTAGATATGGCTATTTTTAGTCTTCATGCAGCCGGAGCTTCATCTATAATGGGATCTATGAATTTCATTACTACCATTTTTAATATGAGAGCCCCAGGAATGACT<br>ATGGATAGAGTTCCTTTGTTTGTATGAGCTGTTTTAATAACAGCTTTTCTTCTTTTATTATCCTTACCTGTTCTTGCTGGAGCTATTACAATGTTATTAACTGACCGTAATTTTAA<br>TACAA

Table S2. *Craspedacusta* mtDNA COI "partial" dataset alignment.

>ESP_OR965077<br>GAACCGCTCTTAGCATGTTGATTCGCTTAGAGCTTTCTGGACCAGGAGCTATGTTAGGAGATGACCAAATTTATAATGTCATCGTCACCGCTCATGCTTTCGTCATGATCTT<br>CTTCTTGGTAATGCCTGTGATGATGGGTGGCTTCGGAAACTGATTCGTCCCTCTATATATAGGAGCTCCAGATATGGCATTTCCTCGATTAAATAACCTCAGTTTTTGGCTTC<br>TTCCTCCCGCCCTCTTTTTGTTATTAGGCTCTGCTTTAGTAGAACAAGGAGCAGGAACAGGTTGAACAGTCTACCCCCCACTTGCTGGAGTTCAAGCTCATTCCGGACCTT<br>CAGTAGACATGGCCATCTTTAGCCTTCATGCGGCAGGTGCTTCCTCCATCATGGGTTCTATGAATTTCATCACTACTATTTTCAACATGAGAGCACCAGGAATGACCATGGA<br>TCGCGTTCCCCTCTTCGTTTGAGCTGTACTCATCACGGCATTCCTTTTAGTTCTCTCTTTGCCCGTCTTAGCTGGAGCCATTACTATGTTACTAACTGATCGTAACTTTAATAC<br>CA
>ITA_Lake_Ceresio_OR965078<br>GAACCGCTCTTAGCATGTTGATTCGCTTAGAGCTTTCTGGACCAGGAGCTATGTTAGGAGATGACCAAATTTATAATGTCATCGTCACCGCTCATGCTTTCGTCATGATCTT<br>CTTCTTGGTAATGCCTGTGATGATGGGTGGCTTCGGAAACTGATTCGTCCCTCTATATATAGGAGCTCCAGATATGGCATTTCCTCGATTAAATAACCTCAGTTTTTGGCTTC<br>TTCCTCCCGCCCTCTTTTTGTTATTAGGCTCTGCTTTAGTAGAACAAGGAGCAGGAACAGGTTGAACAGTCTACCCCCCACTTGCTGGAGTTCAAGCTCATTCCGGACCTT<br>CAGTAGACATGGCCATCTTTAGCCTTCATGCGGCAGGTGCTTCCTCCATCATGGGTTCTATGAATTTCATCACTACTATTTTCAACATGAGAGCACCAGGAATGACCATGGA<br>TCGCGTTCCCCTCTTCGTTTGAGCTGTACTCATCACGGCATTCCTTTTAGTTCTCTCTTTGCCCGTCTTAGCTGGAGCCATTACTATGTTACTAACTGATCGTAACTTTAATAC<br>CA
>ITA_Lake_Ceresio_OR965079<br>GAACCGCTCTTAGCATGTTGATTCGCTTAGAGCTTTCTGGACCAGGAGCTATGTTAGGAGATGACCAAATTTATAATGTCATCGTCACCGCTCATGCTTTCGTCATGATCTT<br>CTTCTTGGTAATGCCTGTGATGATGGGTGGCTTCGGAAACTGATTCGTCCCTCTATATATAGGAGCTCCAGATATGGCATTTCCTCGATTAAATAACCTCAGTTTTTGGCTTC<br>TTCCTCCCGCCCTCTTTTTGTTATTAGGCTCTGCTTTAGTAGAACAAGGAGCAGGAACAGGTTGAACAGTCTACCCCCCACTTGCTGGAGTTCAAGCTCATTCCGGACCTT<br>CAGTAGACATGGCCATCTTTAGCCTTCATGCGGCAGGTGCTTCCTCCATCATGGGTTCTATGAATTTCATCACTACTATTTTCAACATGAGAGCACCAGGAATGACCATGGA<br>TCGCGTTCCCCTCTTCGTTTGAGCTGTACTCATCACGGCATTCCTTTTAGTTCTCTCTTTGCCCGTCTTAGCTGGAGCCATTACTATGTTACTAACTGATCGTAACTTTAATAC<br>CA
>ITA_Large_Lake_Monticolo_OR965080<br>GAACCGCTCTTAGCATGTTGATTCGCTTAGAGCTTTCTGGACCAGGAGCTATGTTAGGAGATGACCAAATTTATAATGTCATCGTCACCGCTCATGCTTTCGTCATGATCTT<br>CTTCTTGGTAATGCCTGTGATGATGGGTGGCTTCGGAAACTGATTCGTCCCTCTATATATAGGAGCTCCAGATATGGCATTTCCTCGATTAAATAACCTCAGTTTTTGGCTTC<br>TTCCTCCCGCCCTCTTTTTGTTATTAGGCTCTGCTTTAGTAGAACAAGGAGCAGGAACAGGTTGAACAGTCTACCCCCCACTTGCTGGAGTTCAAGCTCATTCCGGACCTT<br>CAGTAGACATGGCCATCTTTAGCCTTCATGCGGCAGGTGCTTCCTCCATCATGGGTTCTATGAATTTCATCACTACTATCTTCAACATGAGAGCACCAGGAATGACCATGG<br>ATCGCGTTCCCCTCTTCGTTTGAGCCGTACTCATCACGGCATTCCTTTTAGTTCTCTCTTTGCCCGTCTTAGCTGGAGCGATTACTATGTTACTAACTGATCGTAACTTTAATA<br>CCA
>ITA_Lake_Endine_OR965081<br>GAACCGCCCTTAGCATGCTTATTCGCTTGGAACTATCGGGACCGGGAGCCATGCTAGGAGACGATCAGATCTACAACGTCATCGTCACTGCTCACGCTTTCGTCATGATTT<br>TCTTCTTGGTCATGCCGGTAATGATGGGTGGATTCGGAAACTGATTCGTCCCTCTTTACATCGGAGCGCCAGACATGGCCTTTCCTCGATTAAACAACCTCAGTTTCTGACT<br>CCTTCCTCCTGCCCTCTTCTTATTGCTTGGGTCGGCTTTAGTGGAACAAGGAGCAGGTACAGGTTGGACGGTCTATCCCCCTCTTGCCGGAGTTCAGGCCCACTCCGGACC<br>TTCCGTGGATATGGCCATCTTTAGCCTTCATGCGGCCGGTGCCTCTTCTATCATGGGATCCATGAACTTCATCACCACCATCTTTAACATGAGAGCCCCAGGGATGACCATG<br>GATCGCGTTCCTCTCTTCGTCTGGGCAGTGCTAATCACGGCCTTCCTTCTGGTTCTATCTCTTCCCGTCTTAGCAGGAGCCATCACCATGCTACTCACGGACCGTAACTTCA<br>ACACCA
>ITA_Lake_Endine_OR965082<br>GAACCGCCCTTAGCATGCTTATTCGCTTGGAACTATCGGGACCGGGAGCCATGCTAGGAGACGATCAGATCTACAACGTCATCGTCACTGCTCACGCTTTCGTCATGATTT<br>TCTTCTTGGTCATGCCGGTAATGATGGGTGGATTCGGAAACTGATTCGTCCCTCTTTACATCGGAGCGCCAGACATGGCCTTTCCTCGATTAAACAACCTCAGTTTCTGACT<br>CCTTCCTCCTGCCCTCTTCTTATTGCTTGGGTCGGCTTTAGTGGAACAAGGAGCAGGTACAGGTTGGACGGTCTATCCCCCTCTTGCCGGAGTTCAGGCCCACTCCGGACC<br>TTCCGTGGATATGGCCATCTTTAGCCTTCATGCGGCCGGTGCCTCTTCTATCATGGGATCCATGAACTTCATCACCACCATCTTTAACATGAGAGCCCCAGGGATGACCATG<br>GATCGCGTTCCTCTCTTCGTCTGGGCAGTGCTAATCACGGCCTTCCTTCTGGTTCTATCTCTTCCCGTCTTAGCAGGAGCCATCACCATGCTACTCACGGACCGTAACTTCA<br>ACACCA
>ITA_Lake_Levico_OR965083<br>GAACCGCCCTTAGCATGCTTATTCGCTTGGAACTATCGGGACCGGGAGCCATGCTAGGAGACGATCAGATCTACAACGTCATCGTCACTGCTCACGCTTTCGTCATGATTT<br>TCTTCTTGGTCATGCCGGTAATGATGGGTGGATTCGGAAACTGATTCGTCCCTCTTTACATCGGAGCGCCAGACATGGCCTTTCCTCGATTAAACAACCTCAGTTTCTGACT<br>CCTTCCTCCTGCCCTCTTCTTATTGCTTGGGTCGGCTTTAGTGGAACAAGGAGCAGGTACAGGTTGGACGGTCTATCCCCCTCTTGCCGGAGTTCAGGCCCACTCCGGACC<br>TTCCGTGGATATGGCCATCTTTAGCCTTCATGCGGCCGGTGCCTCTTCTATCATGGGATCCATGAACTTCATCACCACCATCTTTAACATGAGAGCCCCAGGGATGACCATG<br>GATCGCGTTCCTCTCTTCGTCTGGGCAGTGCTAATCACGGCCTTCCTTCTGGTTCTATCTCTTCCCGTCTTAGCAGGAGCCATCACCATGCTACTCACGGACCGTAACTTCA<br>ACACCA

>ITA_Lake_Levico_OR965084<br>GAACCGCCCTTAGCATGCTTATTCGCTTGGAACTATCGGGACCGGGAGCCATGCTAGGAGACGATCAGATCTACAACGTCATCGTCACTGCTCACGCTTTCGTCATGATTT<br>TCTTCTTGGTCATGCCGGTAATGATGGGTGGATTCGGAAACTGATTCGTCCCTCTTTACATCGGAGCGCCAGACATGGCCTTTCCTCGATTAAACAACCTCAGTTTCTGACT<br>CCTTCCTCCTGCCCTCTTCTTATTGCTTGGGTCGGCTTTAGTGGAACAAGGAGCAGGTACAGGTTGGACGGTCTATCCCCCTCTTGCCGGAGTTCAGGCCCACTCCGGACC<br>TTCCGTGGATATGGCCATCTTTAGCCTTCATGCGGCCGGTGCCTCTTCTATCATGGGATCCATGAACTTCATCACCACCATCTTTAACATGAGAGCCCCAGGGATGACCATG<br>GATCGCGTTCCTCTCTTCGTCTGGGCAGTGCTAATCACGGCCTTCCTTCTGGTTCTATCTCTTCCCGTCTTAGCAGGAGCCATCACCATGCTACTCACGGACCGTAACTTCA<br>ACACCA
>ITA_Lake_Santo_of_Monte_Terlago_OR965085<br>GAACCGCCCTTAGCATGCTTATTCGCTTGGAACTATCGGGACCGGGAGCCATGCTAGGAGACGATCAGATCTACAACGTCATCGTCACTGCTCACGCTTTCGTCATGATTT<br>TCTTCTTGGTCATGCCGGTAATGATGGGTGGATTCGGAAACTGATTCGTCCCTCTTTACATCGGAGCGCCAGACATGGCCTTTCCTCGATTAAACAACCTCAGTTTCTGACT<br>CCTTCCTCCTGCCCTCTTCTTATTGCTTGGGTCGGCTTTAGTGGAACAAGGAGCAGGTACAGGTTGGACGGTCTATCCCCCTCTTGCCGGAGTTCAGGCCCACTCCGGACC<br>TTCCGTGGATATGGCCATCTTTAGCCTTCATGCGGCCGGTGCCTCTTCTATCATGGGATCCATGAACTTCATCACCACCATCTTTAACATGAGAGCCCCAGGGATGACCATG<br>GATCGCGTTCCTCTCTTCGTCTGGGCAGTGCTAATCACGGCCTTCCTTCTGGTTCTATCTCTTCCCGTCTTAGCAGGAGCCATCACCATGCTACTCACGGACCGTAACTTCA<br>ACACCA
>ITA_Lake_Santo_of_Monte_Terlago_OR965086<br>GAACCGCCCTTAGCATGCTTATTCGCTTGGAACTATCGGGACCGGGAGCCATGCTAGGAGACGATCAGATCTACAACGTCATCGTCACTGCTCACGCTTTCGTCATGATTT<br>TCTTCTTGGTCATGCCGGTAATGATGGGTGGATTCGGAAACTGATTCGTCCCTCTTTACATCGGAGCGCCAGACATGGCCTTTCCTCGATTAAACAACCTCAGTTTCTGACT<br>CCTTCCTCCTGCCCTCTTCTTATTGCTTGGGTCGGCTTTAGTGGAACAAGGAGCAGGTACAGGTTGGACGGTCTATCCCCCTCTTGCCGGAGTTCAGGCCCACTCCGGACC<br>TTCCGTGGATATGGCCATCTTTAGCCTTCATGCGGCCGGTGCCTCTTCTATCATGGGATCCATGAACTTCATCACCACCATCTTTAACATGAGAGCCCCAGGGATGACCATG<br>GATCGCGTTCCTCTCTTCGTCTGGGCAGTGCTAATCACGGCCTTCCTTCTGGTTCTATCTCTTCCCGTCTTAGCAGGAGCCATCACCATGCTACTCACGGACCGTAACTTCA<br>ACACCA
>ITA_Reservoir_Palermo_MH230079<br>GAACCGCCCTTAGCATGCTTATTCGCTTGGAACTATCGGGACCGGGAGCCATGCTAGGAGACGATCAGATCTACAACGTCATCGTCACTGCTCACGCTTTCGTCATGATTT<br>TCTTCTTGGTCATGCCGGTAATGATGGGTGGATTCGGAAACTGATTCGTCCCTCTTTACATCGGAGCGCCAGACATGGCCTTTCCTCGATTAAACAACCTCAGTTTCTGACT<br>CCTTCCTCCTGCCCTCTTCTTATTGCTTGGGTCGGCTTTAGTGGAACAAGGAGCAGGTACAGGTTGGACGGTCTATCCCCCTCTTGCCGGAGTTCAGGCCCACTCCGGACC<br>TTCCGTGGATATGGTCATCTTTAGCCTTCATGCGGCCGGTGCCTCTTCTATCATGGGATCCATGAACTTCATCACCACCATCTTTAACATGAGAGCCCCAGGGATGACCATG<br>GATCGCGTTCCTCTCTTCGTCTGGGCAGTGCTAATCACGGCCTTCCTTCTGGTTCTATCTCTTCCCGTCTTAGCAGGAGCCATCACCATGCTACTCACGGACCGTAACTTCA<br>ACACCA
>DEU_MZ508274<br>GAACCGCTCTTAGCATGTTGATTCGCTTAGAGCTTTCTGGACCAGGAGCTATGTTAGGAGATGACCAAATTTATAATGTCATCGTCACCGCTCATGCTTTCGTCATGATCTT<br>CTTCTTGGTAATGCCTGTGATGATGGGTGGCTTCGGAAACTGATTCGTCCCTCTATATATAGGAGCTCCAGATATGGCATTTCCTCGATTAAATAACCTCAGTTTTTGGCTTC<br>TTCCTCCCGCCCTCTTTTTGTTATTAGGCTCTGCTTTAGTAGAACAAGGAGCAGGAACAGGTTGAACAGTCTACCCCCCACTTGCTGGAGTTCAAGCTCATTCCGGACCTT<br>CAGTAGACATGGCCATCTTTAGCCTTCATGCGGCAGGTGCTTCCTCCATCATGGGTTCTATGAATTTCATCACTACTATCTTCAACATGAGAGCACCAGGAATGACCATGG<br>ATCGCGTTCCCCTCTTCGTTTGAGCCGTACTCATCACGGCATTCCTTTTAGTTCTCTCTTTGCCCGTCTTAGCTGGAGCGATTACTATGTTACTAACTGATCGTAACTTTAATA<br>CCA
>IND_OP120942<br>GAACCGCTCTTAGCATGTTGATTCGCTTAGAGCTTTCTGGACCAGGAGCTATGTTAGGAGATGACCAAATTTATAATGTCATCGTCACCGCTCATGCTTTCGTCATGATCTT<br>CTTCTTGGTAATGCCTGTGATGATGGGTGGCTTCGGAAACTGATTCGTCCCTCTATATATAGGAGCTCCAGATATGGCATTTCCTCGATTAAATAACCTCAGTTTTTGGCTTC<br>TTCCTCCCGCCCTCTTTTTGTTATTAGGCTCTGCTTTAGTAGAACAAGGAGCAGGAACAGGTTGAACAGTCTACCCCCCACTTGCTGGAGTTCAAGCTCATTCCGGACCTT<br>CAGTAGACATGGCCATCTTTAGCCTTCATGCGGCAGGTGCTTCCTCCATCATGGGTTCTATGAATTTCATCACTACTATCTTCAACATGAGAGCACCAGGAATGACCATGG<br>ATCGCGTTCCCCTCTTCGTTTGAGCTGTACTCATCACGGCATTCCTTTTAGTTCTCTCTTTGCCCGTCTTAGCTGGAGCCATTACTATGTTACTAACTGATCGTAACTTTAATA<br>CCA
>DEU_FJ423619<br>GAACCGCTCTTAGCATGTTGATTCGCTTAGAGCTTTCTGGACCAGGAGCTATGTTAGGAGATGACCAAATTTATAATGTCATCGTCACCGCTCATGCTTTCGTCATGATCTT<br>CTTCTTGGTAATGCCTGTGATGATGGGTGGCTTCGGAAACTGATTCGTCCCTCTATATATAGGAGCTCCAGATATGGCATTTCCTCGATTAAATAACCTCAGTTTTTGGCTTC<br>TTCCTCCCGCCCTCTTTTTGTTATTAGGCTCTGCTTTAGTAGAACAAGGAGCAGGAACAGGTTGAACAGTCTACCCCCCACTTGCTGGAGTTCAAGCTCATTCCGGACCTT<br>CAGTAGACATGGCCATCTTTAGCCTTCATGCGGCAGGTGCTTCCTCCATCATGGGTTCTATGAATTTCATCACTACTATTTTCAACATGAGAGCACCAGGAATGACCATGGA<br>TCGCGTTCCCCTCTTCGTTTGAGCTGTACTCATCACGGCATTCCTTTTAGTTCTCTCTTTGCCCGTCTTAGCTGGAGCCATTACTATGTTACTAACTGATCGTAACTTTAATAC<br>CA
>MAR_MK600509<br>GAACCGCTCTTAGCATGTTGATTCGCTTAGAGCTTTCTGGACCAGGAGCTATGTTAGGAGATGACCAAATTTATAATGTCATCGTCACCGCTCATGCTTTCGTCATGATCTT<br>CTTCTTGGTAATGCCTGTGATGATGGGTGGCTTCGGAAACTGATTCGTCCCTCTATATATAGGAGCTCCAGATATGGCATTTCCTCGATTAAATAACCTCAGTTTTTGGCTTC<br>TTCCTCCCGCCCTCTTTTTGTTATTAGGCTCTGCTTTAGTAGAACAAGGAGCAGGAACAGGTTGAACAGTCTACCCCCCACTTGCTGGAGTTCAAGCTCATTCCGGACCTT<br>CAGTAGACATGGCCATCTTTAGCCTTCATGCGGCAGGTGCTTCCTCCATCATGGGTTCTATGAATTTCATCACTACTATTTTCAACATGAGAGCACCAGGAATGACCATGGA<br>TCGCGTTCCCCTCTTCGTTTGAGCTGTACTCATCACGGCATTCCTTTTAGTTCTCTCTTTGCCCGTCTTAGCTGGAGCCATTACTATGTTACTAACTGATCGTAACTTTAATAC<br>CA
>MAR_MK600508<br>GAACCGCTCTTAGCATGTTGATTCGCTTAGAGCTTTCTGGACCAGGAGCTATGTTAGGAGATGACCAAATTTATAATGTCATCGTCACCGCTCATGCTTTCGTCATGATCTT<br>CTTCTTGGTAATGCCTGTGATGATGGGTGGCTTCGGAAACTGATTCGTCCCTCTATATATAGGAGCTCCAGATATGGCATTTCCTCGATTAAATAACCTCAGTTTTTGGCTTC<br>TTCCTCCCGCCCTCTTTTTGTTATTAGGCTCTGCTTTAGTAGAACAAGGAGCAGGAACAGGTTGAACAGTCTACCCCCCACTTGCTGGAGTTCAAGCTCATTCCGGACCTT<br>CAGTAGACATGGCCATCTTTAGCCTTCATGCGGCAGGTGCTTCCTCCATCATGGGTTCTATGAATTTCATCACTACTATTTTCAACATGAGAGCACCAGGAATGACCATGGA<br>TCGCGTTCCCCTCTTCGTTTGAGCTGTACTCATCACGGCATTCCTTTTAGTTCTCTCTTTGCCCGTCTTAGCTGGAGCCATTACTATGTTACTAACTGATCGTAACTTTAATAC<br>CA
>CHN_KF510026<br>GAACCGCTCTTAGCATGTTGATTCGCTTAGAGCTTTCTGGACCAGGAGCTATGTTAGGAGATGACCAAATTTATAATGTCATCGTCACCGCTCATGCTTTCGTCATGATCTT<br>CTTCTTGGTAATGCCTGTGATGATGGGTGGCTTCGGAAACTGATTCGTCCCTCTATATATAGGAGCTCCAGATATGGCATTTCCTCGATTAAATAACCTCAGTTTTTGGCTTC<br>TTCCTCCCGCCCTCTTTTTGTTATTAGGCTCTGCTTTAGTAGAACAAGGAGCAGGAACAGGTTGAACAGTCTACCCCCCACTTGCTGGAGTTCAAGCTCATTCCGGACCTT<br>CAGTAGACATGGCCATCTTTAGCCTTCATGCGGCAGGTGCTTCCTCCATCATGGGTTCTATGAATTTCATCACTACTATCTTCAACATGAGAGCACCAGGAATGACCATGG<br>ATCGCGTTCCCCTCTTCGTTTGAGCTGTACTCATCACGGCATTCCTTTTAGTTCTCTCTTTGCCCGTCTTAGCTGGAGCCATTACTATGTTACTAACTGATCGTAACTTTAATA<br>CCA
>CHE_MF000493<br>GAACCGCTCTCAGTATGCTCATTCGCTTAGAGCTCTCTGGACCCGGAGCCATGTTAGGAGACGACCAAATCTATAACGTCATCGTCACCGCTCACGCTTTCGTCATGATCT<br>TCTTTTTGGTGATGCCCGTGATGATGGGTGGGTTCGGAAACTGATTCGTCCCTCTATACATAGGAGCCCCGGATATGGCTTTCCCTCGATTAAACAATCTCAGTTTCTGACTT<br>CTTCCTCCCGCCCTATTCCTGTTACTCGGTTCTGCTTTGGTAGAACAAGGAGCAGGTACGGGTTGAACAGTTTATCCCCCACTTGCCGGGGTTCAAGCTCATTCTGGCCCCT<br>CGGTAGACATGGCCATCTTCAGTCTTCATGCGGCAGGTGCTTCCTCGATCATGGGTTCCATGAACTTCATCACCACCATCTTTAACATGAGAGCCCCAGGAATGACGATGG<br>ATCGCGTACCTCTGTTCGTGTGAGCCGTACTCATCACAGCCTTCCTTTTAGTTCTGTCTTTACCCGTTTTAGCTGGAGCCATTACCATGTTGTTGACAGATCGTAATTTCAATA<br>CGA
>Unknown_MW543029<br>GAACCGCCCTTAGCATGCTTATTCGCTTGGAACTATCGGGACCGGGAGCCATGCTAGGAGACGATCAGATCTACAACGTCATCGTCACTGCTCACGCTTTCGTCATGATTT<br>TCTTCTTGGTCATGCCGGTAATGATGGGTGGATTCGGAAACTGATTCGTCCCTCTTTACATCGGAGCGCCAGACATGGCTTTTCCTCGATTAAATAACCTCAGTTTCTGGCT<br>CCTCCCTCCTGCCCTCTTCTTATTGCTTGGATCGGCTTTAGTGGAACAAGGAGCAGGTACAGGTTGGACGGTCTATCCCCCTCTTGCCGGAGTTCAGGCTCACTCCGGACC<br>TTCCGTGGATATGGCCATCTTTAGCCTTCATGCGGCCGGTGCCTCCTCTATCATGGGATCCATGAACTTCATCACCACCATCTTTAACATGAGAGCCCCAGGGATGACCATG<br>GATCGCGTTCCTCTCTTCGTCTGGGCAGTACTAATCACGGCCTTCCTTCTGGTTCTATCTCTCCCCGTCTTAGCAGGGGCCATCACCATGCTACTCACGGACCGTAACTTCA<br>ACACCA
>CAN_MW600236<br>GAACCGCCCTTAGCATGCTTATTCGCTTGGAACTATCGGGACCGGGAGCCATGCTAGGAGACGATCAGATCTACAACGTCATCGTCACTGCTCACGCTTTCGTCATGATTT<br>TCTTCTTGGTCATGCCGGTAATGATGGGTGGATTCGGAAACTGATTCGTCCCTCTTTACATCGGAGCGCCAGACATGGCCTTTCCTCGATTAAACAACCTCAGTTTCTGACT

TCTTCCCCCTGCCCTCTTCTTATTGCTTGGGTCGGCTTTAGTGGAACAAGGAGCAGGTACAGGTTGGACGGTCTATCCCCCTCTTGCCGGAGTTCAGGCCCACTCCGGACC<br>TTCCGTGGATATGGCCATCTTTAGCCTTCATGCGGCCGGTGCCTCTTCTATCATGGGATCCATGAACTTCATCACCACCATCTTTAACATGAGAGCCCCAGGGATGACCATG<br>GATCGCGTTCCTCTCTTCGTCTGAGCAGTGCTAATCACGGCCTTCCTTCTGGTTCTATCTCTTCCCGTCTTAGCAGGAGCCATCACCATGCTACTCACGGACCGTAACTTCA<br>ACACCA
>CAN_ON606485<br>GAACCGCCCTTAGCATGCTTATTCGCTTGGAACTATCGGGACCGGGAGCCATGCTAGGAGACGATCAGATCTACAACGTCATCGTCACTGCTCACGCTTTCGTCATGATTT<br>TCTTCTTGGTCATGCCGGTAATGATGGGTGGATTCGGAAACTGATTCGTCCCTCTTTACATCGGAGCGCCAGACATGGCCTTTCCTCGATTAAACAACCTCAGTTTCTGACT<br>TCTTCCCCCTGCCCTCTTCTTATTGCTTGGGTCGGCTTTAGTGGAACAAGGAGCAGGTACAGGTTGGACGGTCTATCCCCCTCTTGCCGGAGTTCAGGCCCACTCCGGACC<br>TTCCGTGGATATGGCCATCTTTAGCCTTCATGCGGCCGGTGCCTCTTCTATCATGGGATCCATGAACTTCATCACCACCATCTTTAACATGAGAGCCCCAGGGATGACCATG<br>GATCGCGTTCCTCTCTTCGTCTGAGCAGTGCTAATCACGGCCTTCCTTCTGGTTCTATCTCTTCCCGTCTTAGCAGGAGCCATCACCATGCTACTCACGGACCGTAACTTCA<br>ACACCA
>SGP_OK037612<br>GAACCGCCCTTAGCATGCTTATTCGCTTGGAACTATCGGGACCGGGAGCCATGCTAGGAGACGATCAGATCTACAACGTCATCGTCACTGCTCACGCTTTCGTCATGATTT<br>TCTTCTTGGTCATGCCGGTAATGATGGGTGGATTCGGAAACTGATTCGTCCCTCTTTACATCGGAGCGCCAGACATGGCCTTTCCTCGATTAAATAACCTCAGTTTCTGACT<br>CCTCCCTCCTGCCCTCTTCTTATTGCTTGGATCGGCTTTAGTGGAACAAGGAGCAGGTACAGGTTGGACGGTCTATCCCCCTCTTGCCGGAGTTCAGGCTCACTCCGGACC<br>TTCCGTGGATATGGCCATCTTTAGCCTTCATGCGGCCGGTGCCTCCTCTATCATGGGATCCATGAACTTCATCACCACCATCTTTAACATGAGAGCCCCAGGGATGACCATG<br>GATCGCGTTCCTCTCTTCGTCTGGGCAGTACTAATCACGGCCTTCCTTCTGGTTCTATCTCTCCCCGTCTTAGCAGGAGCCATCACCATGCTACTCACGGACCGTAACTTCA<br>ACACCA
>JPN_OK037607<br>GAACCGCCCTTAGCATGCTTATTCGCTTGGAACTATCGGGACCGGGAGCCATGCTAGGAGACGATCAGATCTACAACGTCATCGTCACTGCTCACGCTTTCGTCATGATTT<br>TCTTCTTGGTCATGCCGGTAATGATGGGTGGATTCGGAAACTGATTCGTCCCTCTTTACATCGGAGCGCCAGACATGGCCTTTCCTCGATTAAACAACCTCAGTTTCTGACT<br>TCTTCCCCCTGCCCTCTTCTTATTGCTTGGGTCGGCTTTAGTGGAACAAGGAGCAGGTACAGGTTGGACGGTCTATCCCCCTCTTGCCGGAGTTCAGGCCCACTCCGGACC<br>TTCCGTGGATATGGCCATCTTTAGCCTTCATGCGGCCGGTGCCTCTTCTATCATGGGATCCATGAACTTCATCACCACCATCTTTAACATGAGAGCCCCAGGGATGACCATG<br>GATCGCGTTCCTCTCTTCGTCTGAGCAGTGCTAATCACGGCCTTCCTTCTGGTTCTATCTCTTCCCGTCTTAGCAGGAGCCATCACCATGCTACTCACGGACCGTAACTTCA<br>ACACCA
>CHN_MT806195<br>GAACCGCCCTTAGCATGCTTATTCGCTTGGAACTATCGGGACCGGGAGCCATGCTAGGAGACGATCAGATCTACAACGTCATCGTCACTGCTCACGCTTTCGTCATGATTT<br>TCTTCTTGGTCATGCCGGTAATGATGGGTGGATTCGGAAACTGATTCGTCCCTCTTTACATCGGAGCGCCAGACATGGCTTTTCCTCGATTAAATAACCTCAGTTTCTGGCT<br>CCTCCCTCCTGCCCTCTTCTTATTGCTTGGATCGGCTTTAGTGGAACAAGGAGCAGGTACAGGTTGGACGGTCTATCCCCCTCTTGCCGGAGTTCAGGCTCACTCCGGACC<br>TTCCGTGGATATGGCCATCTTTAGCCTTCATGCGGCCGGTGCCTCCTCTATCATGGGATCCATGAACTTCATCACCACCATCTTTAACATGAGAGCCCCAGGGATGACCATG<br>GATCGCGTTCCTCTCTTCGTCTGGGCAGTACTAATCACGGCCTTCCTTCTGGTTCTATCTCTCCCCGTCTTAGCAGGGGCCATCACCATGCTACTCACGGACCGTAACTTCA<br>ACACCA
>JPN_OK037606<br>GAACCGCCCTTAGCATGCTTATTCGCTTGGAACTATCGGGACCGGGAGCCATGCTAGGAGACGATCAGATCTACAACGTCATCGTCACTGCTCACGCTTTCGTCATGATTT<br>TCTTCTTGGTCATGCCGGTAATGATGGGTGGATTCGGAAACTGATTCGTCCCTCTTTACATCGGAGCGCCAGACATGGCCTTTCCTCGATTAAACAACCTCAGTTTCTGACT<br>TCTTCCCCCTGCCCTCTTCTTATTGCTTGGGTCGGCTTTAGTGGAACAAGGAGCAGGTACAGGTTGGACGGTCTATCCCCCTCTTGCCGGAGTTCAGGCCCACTCCGGACC<br>TTCCGTGGATATGGCCATCTTTAGCCTTCATGCGGCCGGTGCCTCTTCTATCATGGGATCCATGAACTTCATCACCACCATCTTTAACATGAGAGCCCCAGGGATGACCATG<br>GATCGCGTTCCTCTCTTCGTCTGAGCAGTGCTAATCACGGCCTTCCTTCTGGTTCTATCTCTTCCCGTCTTAGCAGGAGCCATCACCATGCTACTCACGGACCGTAACTTCA<br>ACACCA
>JPN_OK037605<br>GAACCGCCCTTAGCATGCTTATTCGCTTGGAACTATCGGGACCGGGAGCCATGCTAGGAGACGATCAGATCTACAACGTCATCGTCACTGCTCACGCTTTCGTCATGATTT<br>TCTTCTTGGTCATGCCGGTAATGATGGGTGGATTCGGAAACTGATTCGTCCCTCTTTACATCGGAGCGCCAGACATGGCCTTTCCTCGATTAAACAACCTCAGTTTCTGACT<br>TCTTCCCCCTGCCCTCTTCTTATTGCTTGGGTCGGCTTTAGTGGAACAAGGAGCAGGTACAGGTTGGACGGTCTATCCCCCTCTTGCCGGAGTTCAGGCCCACTCCGGACC<br>TTCCGTGGATATGGCCATCTTTAGCCTTCATGCGGCCGGTGCCTCTTCTATCATGGGATCCATGAACTTCATCACCACCATCTTTAACATGAGAGCCCCAGGGATGACCATG<br>GATCGCGTTCCTCTCTTCGTCTGAGCAGTGCTAATCACGGCCTTCCTTCTGGTTCTATCTCTTCCCGTCTTAGCAGGAGCCATCACCATGCTACTCACGGACCGTAACTTCA<br>ACACCA
>CHN_MT884060<br>GAACCGCCCTTAGCATGCTTATTCGCTTGGAACTATCGGGACCGGGAGCCATGCTAGGAGACGATCAGATCTACAACGTCATCGTCACTGCTCACGCTTTCGTCATGATTT<br>TCTTTTTGGTCATGCCGGTAATGATGGGTGGATTCGGAAACTGATTCGTCCCTCTTTACATCGGAGCGCCAGACATGGCCTTTCCTCGATTAAACAACCTCAGTTTCTGACT<br>CCTTCCTCCTGCCCTCTTCTTATTGCTTGGGTCGGCTTTAGTGGAACAAGGAGCAGGTACAGGTTGGACGGTCTATCCCCCTCTTGCCGGAGTTCAGGCCCACTCCGGACC<br>TTCCGTGGATATGGCCATCTTTAGCCTTCATGCGGCCGGTGCCTCTTCTATCATGGGATCCATGAACTTCATCACCACCATCTTTAACATGAGAGCCCCAGGGATGACCATG<br>GATCGCGTTCCTCTCTTCGTCTGGGCAGTGCTAATCACGGCCTTCCTTCTGGTTCTATCTCTTCCCGTCTTGGCAGGAGCCATCACCATGCTACTCACGGACCGTAACTTCA<br>ACACCA
>GRE_KP231217<br>GAACCGCCCTTAGCATGCTTATTCGCTTGGAACTATCGGGACCGGGAGCCATGCTAGGAGACGATCAGATCTACAACGTTATCGTCACTGCTCACGCTTTCGTCATGATTT<br>TCTTCTTGGTCATGCCGGTAATGATGGGTGGATTCGGAAACTGATTCGTCCCTCTTTACATCGGAGCGCCAGACATGGCCTTTCCTCGATTAAATAACCTCAGTTTCTGACT<br>CCTCCCTCCTGCCCTCTTCTTATTGCTTGGATCGGCTTTAGTGGAACAAGGAGCAGGTACAGGTTGGACGGTCTATCCCCCTCTTGCCGGAGTTCAGGCTCACTCCGGACC<br>TTCCGTGGATATGGCCATCTTTAGCCTTCATGCGGCCGGTGCCTCCTCTATCATGGGATCCATGAACTTCATCACCACCATCTTTAACATGAGAGCCCCAGGGATGACCATG<br>GATCGCGTTCCTCTCTTCGTCTGGGCAGTACTAATCACGGCCTTCCTTCTGGTTCTATCTCTCCCCGTCTTAGCAGGAGCCATCACCATACTACTCACGGACCGTAACTTCA<br>ACACCA
>DEU_FJ423613<br>GAACCGCTCTTAGCATGTTGATTCGCTTAGAGCTTTCTGGACCAGGAGCTATGTTAGGAGATGACCAAATTTATAATGTCATCGTCACCGCTCATGCTTTCGTCATGATCTT<br>CTTCTTGGTAATGCCTGTGATGATGGGTGGCTTCGGAAACTGATTCGTCCCTCTATATATAGGAGCTCCAGATATGGCATTTCCTCGATTAAATAACCTCAGTTTTTGGCTTC<br>TTCCTCCCGCCCTCTTTTTGTTATTAGGCTCTGCTTTAGTAGAACAAGGAGCAGGAACAGGTTGAACAGTCTACCCCCCACTTGCTGGAGTTCAAGCTCATTCCGGACCTT<br>CAGTAGACATGGCCATCTTTAGCCTTCATGCGGCAGGTGCTTCCTCCATCATGGGTTCTATGAATTTCATCACTACTATTTTCAACATGAGAGCACCAGGAATGACCATGGA<br>TCGCGTTCCCCTCTTCGTTTGAGCTGTACTCATCACGGCATTCCTTTTAGTTCTCTCTTTGCCCGTCTTAGCTGGAGCCATTACTATGTTACTAACTGATCGTAACTTTAATAC<br>CA
>JPN_MZ326744<br>GAACCGCTCTCAGTATGCTCATTCGCTTAGAGCTCTCTGGACCCGGAGCCATGTTAGGAGACGACCAAATCTATAACGTCATCGTCACCGCTCACGCTTTCGTCATGATCT<br>TCTTTTTGGTGATGCCCGTGATGATGGGTGGGTTCGGAAACTGATTCGTCCCTCTATACATAGGAGCCCCGGATATGGCTTTCCCTCGATTAAACAATCTCAGTTTCTGACTT<br>CTTCCTCCCGCCCTATTCCTGTTACTCGGTTCTGCTTTGGTAGAACAAGGAGCAGGTACGGGTTGAACAGTTTATCCCCCACTTGCCGGGGTTCAAGCTCATTCTGGCCCCT<br>CGGTAGACATGGCCATCTTCAGTCTTCATGCGGCAGGTGCTTCCTCGATCATGGGTTCCATGAACTTCATCACCACCATCTTTAACATGAGAGCCCCAGGAATGACGATGG<br>ATCGCGTACCTCTGTTCGTGTGAGCCGTACTCATCACAGCCTTCCTTTTAGTTCTGTCTTTACCCGTTTTAGCTGGAGCCATTACCATGTTGTTGACAGATCGTAATTTCAATA<br>CGA
>Unknown_MZ146922<br>GAACCGCTCTCAGTATGCTCATTCGCTTAGAGCTCTCTGGACCCGGAGCCATGTTAGGAGACGACCAAATCTATAACGTCATCGTCACCGCTCACGCTTTCGTCATGATCT<br>TCTTTTTGGTGATGCCCGTGATGATGGGTGGGTTCGGAAACTGATTCGTCCCTCTATACATAGGAGCCCCGGATATGGCTTTCCCTCGATTAAACAATCTCAGTTTCTGACTT<br>CTTCCTCCCGCCCTATTCTTGTTACTCGGTTCTGCTCTGGTAGAACAAGGAGCAGGTACGGGTTGAACAGTTTATCCCCCACTTGCCGGGGTTCAAGCTCATTCTGGCCCCT<br>CGGTAGACATGGCCATCTTCAGTCTTCATGCGGCAGGTGCTTCCTCGATCATGGGTTCCATGAACTTCATCACCACCATCTTTAACATGAGAGCCCCAGGAATGACGATGG<br>ATCGCGTACCTCTGTTCGTGTGAGCCGTACTCATCACAGCCTTCCTTTTAGTTCTGTCTTTACCCGTTTTAGCTGGAGCCATTACCATGTTGTTGACAGATCGTAATTTCAATA<br>CGA
>Unknown_MZ569028<br>GGACCGCTCTTAGCATGCTGATTCGTCTAGAGCTCTCGGGACCAGGGGCCATGTTAGGAGACGACCAGATCTACAACGTCATCGTCACCGCCCACGCCTTCGTCATGATC<br>TTCTTCTTGGTCATGCCGGTAATGATGGGTGGATTCGGAAACTGATTCGTCCCTCTCTACATCGGAGCACCAGACATGGCTTTTCCTCGATTAAACAATCTCAGTTTCTGAC<br>TTCTACCTCCCGCCCTCTTCTTATTGCTTGGATCGGCTCTAGTAGAACAAGGAGCAGGAACGGGTTGGACGGTCTATCCCCCTCTCGCCGGGGTTCAGGCTCATTCCGGGC<br>CTTCGGTAGACATGGCCATCTTCAGCCTTCACGCGGCTGGTGCCTCCTCTATCATGGGATCCATGAATTTCATCACCACCATCTTTAACATGAGAGCCCCAGGGATGACGA

TGGATCGAGTCCCTCTCTTCGTTTGAGCAGTGCTAATCACGGCCTTTCTGTTAGTCCTCTCTCTACCCGTTTTAGCGGGGGCCATCACCATGTTGCTCACGGATCGTAATTTC<br>AACACCA
>Unknown_MZ569031<br>GAACCGCCCTTAGCATGCTTATTCGCTTGGAACTATCGGGACCGGGAGCCATGCTAGGAGACGATCAGATCTACAACGTCATCGTCACTGCTCACGCTTTCGTCATGATTT<br>TCTTCTTGGTCATGCCGGTAATGATGGGTGGATTCGGAAACTGATTCGTCCCTCTTTACATCGGAGCGCCAGACATGGCTTTTCCTCGATTAAATAACCTCAGTTTCTGGCT<br>CCTCCCTCCTGCCCTCTTCTTATTGCTTGGATCGGCTTTAGTGGAACAAGGAGCAGGTACAGGTTGGACGGTCTATCCCCCTCTTGCCGGAGTTCAGGCTCACTCCGGACC<br>TTCCGTGGATATGGCCATCTTTAGCCTTCATGCGGCCGGTGCCTCCTCTATCATGGGATCCATGAACTTCATCACCACCATCTTTAACATGAGAGCCCCAGGGATGACCATG<br>GATCGCGTTCCTCTCTTCGTCTGGGCAGTACTAATCACGGCCTTCCTTCTGGTTCTATCTCTCCCCGTCTTAGCAGGGGCCATCACCATGCTACTCACGGACCGTAACTTCA<br>ACACCA
>Unknown_MZ569029<br>GAACCGCCCTTAGCATGCTTATTCGCTTGGAACTATCGGGACCGGGAGCCATGCTAGGAGACGATCAGATCTACAACGTCATCGTCACTGCTCACGCTTTCGTCATGATTT<br>TCTTCTTGGTCATGCCGGTAATGATGGGTGGATTCGGAAACTGATTCGTCCCTCTTTACATCGGAGCGCCAGACATGGCTTTTCCTCGATTAAATAACCTCAGTTTCTGGCT<br>CCTCCCTCCTGCCCTCTTCTTATTGCTTGGATCGGCTTTAGTGGAACAAGGAGCAGGTACAGGTTGGACGGTCTATCCCCCTCTTGCCGGAGTTCAGGCTCACTCCGGACC<br>TTCCGTGGATATGGCCATCTTTAGCCTTCATGCGGCCGGTGCCTCCTCTATCATGGGATCCATGAACTTCATCACCACCATCTTTAACATGAGAGCCCCAGGGATGACCATG<br>GATCGCGTTCCTCTCTTCGTCTGGGCAGTACTAATCACGGCCTTCCTTCTGGTTCTATCTCTCCCCGTCTTAGCAGGGGCCATCACCATGCTACTCACGGACCGTAACTTCA<br>ACACCA
>Unknown_MZ569027<br>GAACCGCCCTTAGCATGCTTATTCGCTTGGAACTATCGGGACCGGGAGCCATGCTAGGAGACGATCAGATCTACAACGTCATCGTCACTGCTCACGCTTTCGTCATGATTT<br>TCTTCTTGGTCATGCCGGTAATGATGGGTGGATTCGGAAACTGATTCGTCCCTCTTTACATCGGAGCGCCAGACATGGCTTTTCCTCGATTAAATAACCTCAGTTTCTGGCT<br>CCTCCCTCCTGCCCTCTTCTTATTGCTTGGATCGGCTTTAGTGGAACAAGGAGCAGGTACAGGTTGGACGGTCTATCCCCCTCTTGCCGGAGTTCAGGCTCACTCCGGACC<br>TTCCGTGGATATGGCCATCTTTAGCCTTCATGCGGCCGGTGCCTCCTCTATCATGGGATCCATGAACTTCATCACCACCATCTTTAACATGAGAGCCCCAGGGATGACCATG<br>GATCGCGTTCCTCTCTTCGTCTGGGCAGTACTAATCACGGCCTTCCTTCTGGTTCTATCTCTCCCCGTCTTAGCAGGGGCCATCACCATGCTACTCACGGACCGTAACTTCA<br>ACACCA
>CZE_MZ508275<br>GAACCGCCCTTAGCATGCTTATTCGCTTGGAACTATCGGGACCGGGAGCCATGCTAGGAGACGATCAGATCTACAACGTCATCGTCACTGCTCACGCTTTCGTCATGATTT<br>TCTTCTTGGTCATGCCGGTAATGATGGGTGGATTCGGAAACTGATTCGTCCCTCTTTACATCGGAGCGCCAGACATGGCCTTTCCTCGATTAAATAACCTCAGTTTCTGACT<br>CCTCCCTCCTGCCCTCTTCTTATTGCTTGGATCGGCTTTAGTGGAACAAGGAGCAGGTACAGGTTGGACGGTCTATCCCCCTCTTGCCGGAGTTCAGGCTCACTCCGGACC<br>TTCCGTGGATATGGCCATCTTTAGCCTTCATGCGGCCGGTGCCTCCTCTATCATGGGATCCATGAACTTCATCACCACCATCTTTAACATGAGAGCCCCAGGGATGACCATG<br>GATCGCGTTCCTCTCTTCGTCTGGGCAGTACTAATCACGGCCTTCCTTCTGGTTCTATCTCTCCCCGTCTTAGCAGGAGCCATCACCATGCTACTCACGGACCGTAACTTCA<br>ACACCA
>CHN_JN593332<br>GAACCGCCCTTAGCATGCTTATTCGCTTGGAACTATCGGGACCGGGAGCCATGCTAGGAGACGATCAGATCTACAACGTCATCGTCACTGCTCACGCTTTCGTCATGATTT<br>TCTTCTTGGTCATGCCGGTAATGATGGGTGGATTCGGAAACTGATTCGTCCCTCTTTACATCGGAGCGCCAGACATGGCCTTTCCTCGATTAAATAACCTCAGTTTCTGACT<br>CCTCCCTCCTGCCCTCTTCTTATTGCTTGGATCGGCTTTAGTGGAACAAGGAGCAGGTACAGGTTGGACGGTCTATCCCCCTCTTGCCGGAGTTCAGGCTCACTCCGGACC<br>TTCCGTGGATATGGCCATCTTTAGCCTTCATGCGGCCGGTGCTTCCTCTATCATGGGATCCATGAACTTCATCACCACCATCTTTAACATGAGAGCCCCAGGGATGGCCATG<br>GATCGCGTTCCTCTCTTCGTCTGGGCAGTACTAATCACGGCCTTCCTTCTGGTTCTATCTCTCCCCGTCTTAGCAGGAGCCATCACCATGCTACTCACGGACCGTAACTTCA<br>ACACCA
>DEU_MZ508276<br>GAACCGCCCTTAGCATGCTTATTCGCTTGGAACTATCGGGACCGGGAGCCATGCTAGGAGACGATCAGATCTACAACGTCATCGTCACTGCTCACGCTTTCGTCATGATTT<br>TCTTCTTGGTCATGCCGGTAATGATGGGTGGATTCGGAAACTGATTCGTCCCTCTTTACATCGGAGCGCCAGACATGGCCTTTCCTCGATTAAACAACCTCAGTTTCTGACT<br>CCTTCCTCCTGCCCTCTTCTTATTGCTTGGGTCGGCTTTAGTGGAACAAGGAGCAGGTACAGGTTGGACGGTCTATCCCCCTCTTGCCGGAGTTCAGGCCCACTCCGGACC<br>TTCCGTGGATATGGCCATCTTTAGCCTTCATGCGGCCGGTGCCTCTTCTATCATGGGATCCATGAACTTCATCACCACCATCTTTAACATGAGAGCCCCAGGGATGACCATG<br>GATCGCGTTCCTCTCTTCGTCTGGGCAGTGCTAATCACGGCCTTCCTTCTGGTTCTATCTCTTCCCGTCTTAGCAGGAGCCATCACCATGCTACTCACGGACCGTAACTTCA<br>ACACCA
>Unknown_MZ569030<br>GAACCGCCCTTAGCATGCTTATTCGCTTGGAACTATCGGGACCGGGAGCCATGCTAGGAGACGATCAGATCTACAACGTCATCGTCACTGCTCACGCTTTCGTCATGATTT<br>TCTTTTTGGTCATGCCGGTAATGATGGGTGGATTCGGAAACTGATTCGTCCCTCTTTACATCGGAGCGCCAGACATGGCCTTTCCTCGATTAAACAACCTCAGTTTCTGACT<br>CCTTCCTCCTGCCCTCTTCTTATTGCTTGGGTCGGCTTTAGTGGAACAAGGAGCAGGTACAGGTTGGACGGTCTATCCCCCTCTTGCCGGAGTTCAGGCCCACTCCGGACC<br>TTCCGTGGATATGGCCATCTTTAGCCTTCATGCGGCCGGTGCCTCTTCTATCATGGGATCCATGAACTTCATCACCACCATCTTTAACATGAGAGCCCCAGGGATGACCATG<br>GATCGCGTTCCTCTCTTCGTCTGGGCAGTGCTAATCACGGCCTTCCTTCTGGTTCTATCTCTTCCCGTCTTGGCAGGAGCCATCACCATGCTACTCACGGACCGTAACTTCA<br>ACACCA
>DEU_MZ508273<br>GAACCGCTCTTAGCATGTTGATTCGCTTAGAGCTTTCTGGACCAGGAGCTATGTTAGGAGATGACCAAATTTATAATGTCATCGTCACCGCTCATGCTTTCGTCATGATCTT<br>CTTCTTGGTAATGCCTGTGATGATGGGTGGCTTCGGAAACTGATTCGTCCCTCTATATATAGGAGCTCCAGATATGGCATTTCCTCGATTAAATAACCTCAGTTTTTGGCTTC<br>TTCCTCCCGCCCTCTTTTTGTTATTAGGCTCTGCTTTAGTAGAACAAGGAGCAGGAACAGGTTGAACAGTCTACCCCCCACTTGCTGGAGTTCAAGCTCATTCCGGACCTT<br>CAGTAGACATGGCCATCTTTAGCCTTCATGCGGCAGGTGCTTCCTCCATCATGGGTTCTATGAATTTCATCACTACTATTTTCAACATGAGAGCACCAGGAATGACCATGGA<br>TCGCGTTCCCCTCTTCGTTTGAGCTGTACTCATCACGGCATTCCTTTTAGTTCTCTCTTTGCCCGTCTTAGCTGGAGCCATTACTATGTTACTAACTGATCGTAACTTTAATAC<br>CA
>USA_ON606476<br>GAACCGCCCTTAGCATGCTTATTCGCTTGGAACTATCGGGACCGGGAGCCATGCTAGGAGACGATCAGATCTACAACGTCATCGTCACTGCTCACGCTTTCGTCATGATTT<br>TCTTCTTGGTCATGCCGGTAATGATGGGTGGATTCGGAAACTGATTCGTCCCTCTTTACATCGGAGCGCCAGACATGGCCTTTCCTCGATTAAACAACCTCAGTTTCTGACT<br>CCTTCCTCCTGCCCTCTTCTTATTGCTTGGGTCGGCTTTAGTGGAACAAGGAGCAGGTACAGGTTGGACGGTCTATCCCCCTCTTGCCGGAGTTCAGGCCCACTCCGGACC<br>TTCCGTGGATATGGCCATCTTTAGCCTTCATGCGGCCGGTGCCTCTTCTATCATGGGATCCATGAACTTCATCACCACCATCTTTAACATGAGAGCCCCAGGGATGACCATG<br>GATCGCGTTCCTCTCTTCGTCTGGGCAGTGCTAATCACGGCCTTCCTTCTGGTTCTATCTCTTCCCGTCTTAGCAGGAGCCATCACCATGCTACTCACGGACCGTAACTTCA<br>ACACCA
>AF383926_Maeotias_marginata<br>GAACAGCTCTAAGCATGCTTATTCGTTTAGAATTATCTGGCCCAGGAGCTATGTTAGGAGATGATCAAATTTATAATGTTATCGTTACCGCTCATGCTTTCGTTATGATTTTCT<br>TCTTAGTTATGCCTGTTTTGATGGGAGGGTTTGGAAATTGATTTGTACCATTATATATAGGTGCACCTGATATGGCTTTTCCAAGATTAAATAATTTAAGTTTTTGATTACTACC<br>TCCTGCTTTATTTTTACTTTTAGGTTCTTCTTTAGTAGAACAAGGTGCTGGAACAGGTTGAACTGTGTATCCACCATTAGCTAGTGTTCAAGCTCATTCAGGACCTTCTGTAG<br>ATATGGCTATTTTTAGTCTTCATGCAGCCGGAGCTTCATCTATAATGGGATCTATGAATTTCATTACTACCATTTTTAATATGAGAGCCCCAGGAATGACTATGGATAGAGTTC<br>CTTTGTTTGTATGAGCTGTTTTAATAACAGCTTTTCTTCTTTTATTATCCTTACCTGTTCTTGCTGGAGCTATTACAATGTTATTAACTGACCGTAATTTTAATACAA
